# Histone variants in archaea and the evolution of combinatorial chromatin complexity

**DOI:** 10.1073/pnas.2007056117

**Published:** 2020-12-07

**Authors:** Kathryn M. Stevens, Jacob B. Swadling, Antoine Hocher, Corinna Bang, Simonetta Gribaldo, Ruth A. Schmitz, Tobias Warnecke

**Affiliations:** ^a^Molecular Systems Group, Quantitative Biology Section, Medical Research Council London Institute of Medical Sciences, London W12 0NN, United Kingdom;; ^b^Institute of Clinical Sciences, Faculty of Medicine, Imperial College London, London W12 0NN, United Kingdom;; ^c^Institute for General Microbiology, University of Kiel, 24118 Kiel, Germany;; ^d^Institute of Clinical Molecular Biology, University of Kiel, 24105 Kiel, Germany;; ^e^Department of Microbiology, Unit “Evolutionary Biology of the Microbial Cell,” Institut Pasteur, 75015 Paris, France

**Keywords:** histone variants, chromatin, archaea, evolution

## Abstract

Nucleosomes in eukaryotes act as platforms for the dynamic integration of epigenetic information. Posttranslational modifications are reversibly added or removed and core histones exchanged for paralogous variants, in concert with changing demands on transcription and genome accessibility. Histones are also common in archaea. Their role in genome regulation, however, and the capacity of individual paralogs to assemble into histone–DNA complexes with distinct properties remain poorly understood. Here, we combine structural modeling with phylogenetic analysis to shed light on archaeal histone paralogs, their evolutionary history, and capacity to generate combinatorial chromatin states through hetero-oligomeric assembly. Focusing on the human commensal *Methanosphaera stadtmanae* as a model archaeal system, we show that the heteromeric complexes that can be assembled from its seven histone paralogs vary substantially in DNA binding affinity and tetramer stability. Using molecular dynamics simulations, we go on to identify unique paralogs in *M. stadtmanae* and *Methanobrevibacter smithii* that are characterized by unstable interfaces between dimers. We propose that these paralogs act as capstones that prevent stable tetramer formation and extension into longer oligomers characteristic of model archaeal histones. Importantly, we provide evidence from phylogeny and genome architecture that these capstones, as well as other paralogs in the Methanobacteriales, have been maintained for hundreds of millions of years following ancient duplication events. Taken together, our findings indicate that at least some archaeal histone paralogs have evolved to play distinct and conserved functional roles, reminiscent of eukaryotic histone variants. We conclude that combinatorially complex histone-based chromatin is not restricted to eukaryotes and likely predates their emergence.

## Significance

Chromatin in eukaryotes is built around histone–DNA complexes, which act as platforms for the integration of regulatory information. Different layers of information are integrated in a combinatorial fashion, for example by exchanging core histones for variants with different properties. We show that histone variants also exist in archaea. In particular, we identify unique archaeal variants that act as capstones, preventing extension of histone–DNA complexes. Importantly, we show that some archaeal histone variants are ancient and have been maintained as distinct units for hundreds of millions of years. Our work suggests that complex combinatorial chromatin that uses histones as its building blocks exists outside eukaryotes and that the ancestor of eukaryotes might have already had complex chromatin.

Cells dynamically regulate access to genomic information in response to upstream signals. This may involve wholesale remodeling of chromatin, for example during spermatogenesis where histones are largely replaced by protamines. Other changes in chromatin state constitute less radical tweaks to preexisting chromatin architecture. In eukaryotes, the nucleosome provides the principal platform for such tweaks, prominently via posttranslational modifications (PTMs) but also through the exchange of core histones for paralogous variants (1). Like PTMs, histone variants can alter nucleosome dynamics or affect the recruitment of *trans* factors to reinforce existing chromatin states, establish new ones, or poise chromatin for future change. In many cases, such paralog exchange is regulated and adaptive. For example, in humans, de novo deposition of one histone variant (H2A.X) and eviction of another (H2A.Z) facilitate repair of ultraviolet-induced double-strand breaks (2).

**Fig. 1.**
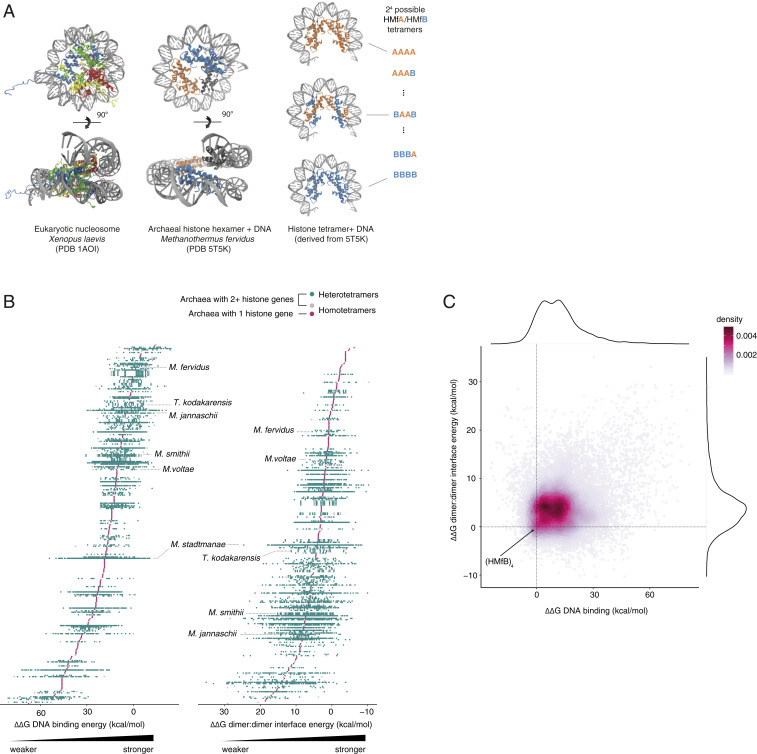
Structural diversity of archaeal histone tetramers. (*A*) Crystal structures of the octameric eukaryotic nucleosome (PDB 1AOI), the hexameric archaeal nucleosome (PDB 5T5K), and the same structure with one dimer removed to yield the tetrameric complex, alongside a schematic showing the different combinations of histones in homo- and heterotetrameric models built for two histones (e.g., *M. fervidus* HMfA and HMfB). (*B*) DNA binding strength and tetramerization strength (dimer:dimer interface energy) for every possible tetrameric histone complex within each species of archaea in our sample. Each point, grouped by species, represents an individual complex. Species are ordered by mean interaction energy across tetramers. Species labels are provided in *SI Appendix*, Fig. S1. (*C*) Relationship between DNA binding and tetramerization strength for each tetrameric model. Most complexes have slightly weaker tetramerization strength and DNA binding than HMfB. ΔΔG is given relative to the HMfB homotetramer for all plots.

Histones are not restricted to eukaryotes but are also common in archaea, where they assemble into tetramers that are structurally very similar to the (H3-H4)_2_ tetramers at the core of eukaryotic nucleosomes (Fig. 1*A*) (3–5). In some archaea, including the model species *Methanothermus fervidus* and *Thermococcus kodakarensis*, additional histone dimers can be tagged onto this tetramer to yield oligomers of increasing length that wrap correspondingly more DNA (3, 6–9). Almost all archaeal histones lack tails and PTMs have yet to be reported. Many archaea do, however, encode multiple histone paralogs (8, 10) that can flexibly homo- and heterodimerize in some species and—in principle—generate chromatin states of considerable combinatorial complexity.

Prior studies in a handful of model species found that archaeal histone paralogs can differ in their expression through the growth cycle, DNA binding affinity, and oligomerization potential, and specific effects on growth and transcription were evident when different paralogs from the same archaeon were deleted (8, 11–14). Yet how the properties of different histone paralogs combine within a single cell to generate dynamic, responsive chromatin states and whether archaeal histone paralogs play conserved roles akin to eukaryotic histone variants remain unknown.

Here, we shed light on the evolution of archaeal histone paralogs and their capacity to generate diverse chromatin states through multimeric assembly. Combining in silico fast mutational scanning with molecular dynamics (MD) simulations and evolutionary analysis, we show that histone paralogs can generate substantial diversity when it comes to key structural properties of the histone–DNA complex. Using *Methanosphaera stadtmanae*—which encodes an unusually large number of histone paralogs (seven)—as a case study, we show that chromatin state space in this multihistone system is large but dense and can be traversed smoothly by altering the dosage of individual paralogs. At the same time, we highlight the potential for more radical change: We describe the widespread existence of capstones—histones that are predicted to prevent further oligomer extension. Importantly, we show that capstones (and other paralogs) in the Methanobacteriales are related by vertical descent, providing evidence for long-term maintenance of functionally distinct paralogs akin to eukaryotic histone variants. Finally, we trace divergent paralog properties to individual amino acid residues and show that paralog diversification has been driven by substitutions at structurally sensitive sites. We propose that paralog exchange might constitute a major mechanism of chromatin state change in archaea, a mechanism that was complemented—and arguably superseded—in eukaryotes by the proliferation of posttranslational modifications. Our results suggest that the last common ancestor of eukaryotes, which emerged from within the Archaea (15, 16), might have already possessed histone-based chromatin of considerable combinatorial complexity, with implications for the contribution of histones to the establishment of eukaryotes (17).

## Results

### Heteromeric Histone–DNA Complexes Exhibit Large Differences in DNA Binding Affinity and Stability across Archaea.

Our current knowledge of functional differences among archaeal histone paralogs is limited, especially for archaea with more than two histone genes, where functional diversity might be greatest. Many of these archaea remain genetically inaccessible and/or difficult to culture, preempting detailed experimental characterization. This includes archaea from the Asgard clade, the closest known relatives of eukaryotes (15, 16). To shed light on the functional diversity of histone paralogs in archaea, we therefore combined structural modeling approaches with evolutionary analysis.

First, using the hexameric crystal structure of HMfB from *M. fervidus* as a template, we built models of tetrameric histone complexes bound to DNA for 282 diverse archaea (*Methods*). Tetramers constitute the minimal oligomeric unit capable of wrapping DNA and have been observed in a range of archaea in vivo (3, 9, 18, 19). For archaea with more than one histone gene, we modeled all possible tetrameric combinations (*n*^4^, where *n* is the number of paralogs; Fig. 1*A*), excluding only histones with large insertions, deletions, or terminal extensions (tails) and those with deletions in the core histone fold (*Methods*). This resulted in 349 homo-oligomeric and 15,905 hetero-oligomeric complexes in total. We then considered Gibbs free energy changes (∆G) at the DNA–protein interface (a measure of DNA binding affinity) and at the interface between the two histone dimers (a measure of tetramer stability; *Methods*). Across our diverse sample of archaea, we observe substantial apparent variability in DNA binding affinity and tetramer stability (Fig. 1 *B* and *C*; *SI Appendix*, Fig. S1; and Dataset S1). Effective differences between species might, however, be less pronounced than they appear. We model tetrameric complexes under standardized conditions (*Methods*), yet archaea differ widely with regard to growth temperature, pH, the concentration of organic and inorganic solutes, and other factors that can influence protein–protein and protein–DNA interactions in vivo. As attempts to systematically control for such potential confounders are plagued by incomplete information, we focus first on comparisons within species, where different heteromeric complexes can be compared more fairly. In particular, we consider *M. stadtmanae*, a mesophilic methanogen that inhabits the human gut, as a case study.

### *M. stadtmanae* as a Case Study for Combinatorially Complex Chromatin.

**Fig. 2.**
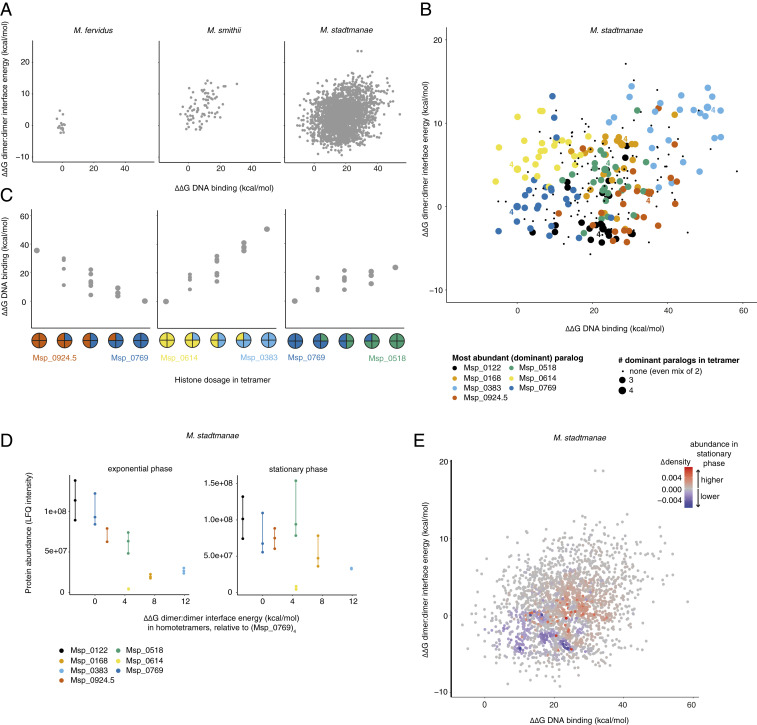
Structural diversity of *M. stadtmanae* tetrameric histone–DNA complexes. (*A*) Chromatin state space defined by DNA binding and tetramerization strength for models built using fast mutational scanning for *M. fervidus* (two histone paralogs), *M. smithii* (three histone paralogs), and *M. stadtmanae* (seven histone paralogs). ΔΔG is given relative to the HMfB homotetramer. (*B*) Chromatin state space defined by DNA binding and tetramerization strength for *M. stadtmanae* histone complexes containing two or fewer histone paralogs. Points are colored by the dominant paralog in the complex (three or four out of four monomers in the tetramer). Homotetramers are labeled (“4”). (*C*) Examples of DNA binding strength varying gradually with paralog dosage. (*D*) Tetramerization strength of *M. stadtmanae* homotetramers compared to empirically determined paralog abundance in exponential and stationary phase. Each data point represents protein abundance measured in one biological replicate. (*E*) Relative change in the abundance of different tetrameric complexes in stationary versus exponential phase, as predicted by sampling 100,000 tetrameric complexes based on relative protein abundance (mean LFQ intensity) in exponential and stationary phase. Increased abundance of complexes in stationary phase is shown in red and decreased abundance in blue. ΔΔG is given relative to Msp_0769 for *B*–*E*.

*M. stadtmanae* DSM3091 encodes seven nonidentical histone genes, located around the chromosome as apparent single-gene operons (*SI Appendix*, Fig. S2). The 7^4^ (= 2,401) tetrameric histone–DNA complexes we built from these histones span the largest DNA affinity range (∆∆G of −10.47 to 54.39 kcal/mol relative to HMfB) and the fourth largest tetramer stability range (∆∆G of −9.33 to 23.63 kcal/mol) in our sample (Figs. 1 *B* and *C* and 2*A*), providing an excellent model system to interrogate the capacity of an individual archaeal cell to generate different chromatin states by altering the composition of histone–DNA complexes via paralog exchange.

We find that tetrameric combinations are not randomly distributed across this state space but occupy partially distinct areas based on which paralog dominates the complex (Fig. 2*B*). Homotetramers are found toward the edges while the intervening space is densely populated (Fig. 2 *A* and *B*). Complexes that are intermediate in terms of paralog dosage tend to have intermediate properties, enabling smooth transitions in chromatin state space, from one extreme to another (Fig. 2 *B* and *C*). Paralogs in this system therefore provide the capacity for graded control of chromatin state through changes in relative paralog dosage, as well as for more radical transitions (below).

### In Vivo Expression of Histone Paralogs in *M. stadtmanae*.

Is the capacity for graded control of chromatin state used dynamically in vivo? And what areas of chromatin state space are actually explored? To begin to address the latter question, we quantified the relative abundance of histone paralogs in exponential and stationary phase *M. stadtmanae* cells using label-free mass spectrometry and RT-qPCR (*Methods* and *SI Appendix*, Fig. S3). Protein abundance varies over a 27-fold range between paralogs but expression levels of individual paralogs are well correlated in exponential and stationary phase (Fig. 2*D* and *SI Appendix*, Fig. S3). Intriguingly, relative paralog abundance in exponential phase exhibits a strong correlation with tetramer stability: Paralogs that are inferred to form more stable homotetramers are more abundant (rho = −0.82, *P* = 0.034; Fig. 2*D*). This is also the case (based on previously determined relative transcript/protein abundance) in *Methanobrevibacter smithii*, another member of the order Methanobacteriales, but not, for example, in the hyperthermophile *T. kodakarensis* (*SI Appendix*, Fig. S4).

To mimic the relative abundance of different complexes in the cell and better approximate actual vis-à-vis theoretical chromatin state space in vivo, we generated 100,000 tetrameric complexes in silico, with individual histones recruited into each complex at random based on their relative abundance at the protein level. Assuming that histones dimerize randomly, we find that the center of gravity in chromatin state space shifts toward complexes that are on average less stable, exhibit lower DNA binding affinity (Fig. 2*E* and *SI Appendix*, Fig. S5), and therefore likely give rise to fewer stable higher-order oligomers. This shift is driven by the up-regulation of two histones, Msp_0168 and Msp_0518 (Fig. 2*D* and *SI Appendix*, Fig. S3), which we infer exhibit relatively low DNA binding affinity and tetramer stability as homotetramers. Thus, we predict that stationary phase should—other things equal—be characterized by more open histone-based chromatin. This again contrasts with prior observations in *M. fervidus*, where expression of HMfB—capable of greater DNA compaction—increases in stationary phase relative to HMfA, the second paralog in *M. fervidus* (11). We return to this difference below.

### MD Simulations of *M. stadtmanae* Homotetramers.

**Fig. 3.**
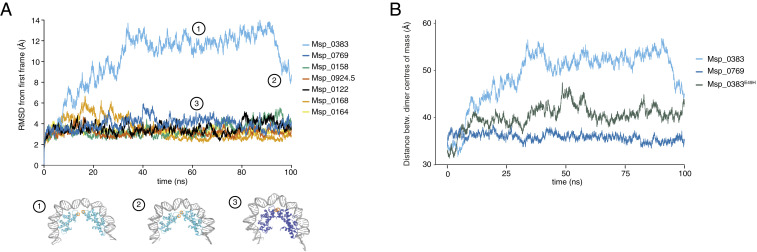
MD simulations of homotetrameric histone models from *M. stadtmanae*. (*A*) Root-mean-square deviation (rmsd) over the course of the simulation, beginning from the crystal structure into which *M. stadtmanae* histones have been substituted (*Methods*). Example structures at different frames are shown for Msp_0383 and Msp_0769 with the position of residue 49 highlighted in orange. (*B*) Distance between the centers of mass of dimers in homotetrameric models of Msp_0383, Msp_0769, and the mutant Msp_0383^E49H^.

To gain more detailed insights into the extremes of *M. stadtmanae* chromatin state space, we carried out extensive MD simulations on all its homotetrameric histone–DNA complexes. DNA binding affinities inferred from these simulations correlate well with results obtained from fast mutational scanning (rho = 0.96, *P* < 0.001; *SI Appendix*, Fig. S6), providing further validation that the fast mutational scanning approach captures salient properties of the histone–DNA complex. The correlation is less tight for tetramerization energies (rho = 0.5, *P* = 0.26), suggestive of dynamic behavior uniquely captured by MD. Indeed, we find that Msp_0383, the paralog with the lowest predicted DNA binding affinity and tetramer stability, exhibits much more extreme spatial displacement from the starting point of the crystal structure than the other histones (Fig. 3*A*). Further analysis of trajectories over the 100-ns simulation revealed that the Msp_0383 homotetramer displays an unstable dimer:dimer interface—unlike the other homomeric complexes, which reach an approximate equilibrium after <20 ns. While the two Msp_0383 dimers remain individually bound to DNA, they are refractory to tetramerization (Fig. 3*A* and Movies S1 and S2). Thus, our modeling predicts that Msp_0383 assembles into histone–DNA complexes that are structurally distinct from classic tetrameric complexes observed for *M. fervidus,* other model archaea, and the remaining *M. stadtmanae* paralogs.

Msp_0383 has a negatively charged glutamic acid (E) at position 49 whereas the other paralogs (and most histones across archaea) have a positively charged histidine (H, Fig. 3*A*) (note that, throughout this article, we number residues based on positional orthology to HMfB; the raw residue number in *M. stadtmanae* is 50). Residue 49 is close to the interface between dimers and mutations at this site were previously shown to impact tetramer formation of HMfB in vitro (18). To test whether amino acid identity at this site is sufficient to account for the repulsive effects observed, we in silico-substituted E for H in all histones of the tetramer and subjected the resulting complex to the same simulation protocol. We find that this substitution alone is enough to significantly reduce the distance between dimers, with Msp_0383^E49H^ exhibiting dynamics that are intermediate between Msp_0383 and the other paralogs (Fig. 3*B*). These results suggest that Msp_0383 functions as a capstone, preventing tetramerization and, when tagged onto an existing complex, further oligomerization.

Such potential capstones are not unique to *M. stadtmanae* but are also present in other members of the Methanobacteriales, as demonstrated by comparative MD simulations of *M. smithii* homotetramers, which also reveal a single, lowly expressed histone (Msm_1260) associated with much-reduced tetramer stability (*SI Appendix*, Figs. S4 and S7).

### Phylogenetic Analysis Reveals Long-Term Persistence of Archaeal Histone Variants.

Some eukaryotic histone variants are ancient and have persisted through multiple rounds of speciation as recognizable, distinct paralogs, often with conserved function and dedicated chaperones that can discriminate between them (20). Notably, this includes H2A.Z, which emerged at the base of eukaryotes. Other variants, like macroH2A, are restricted to certain clades and therefore evolved more recently. Yet others, like H2A.X, appear polyphyletic in origin, pointing to repeated independent emergence of functionally analogous variants (5, 20). What is the situation for archaeal histones? Are there persistent, recognizable paralogs of ancient origin? Or is most diversification relatively recent and lineage specific?

**Fig. 4.**
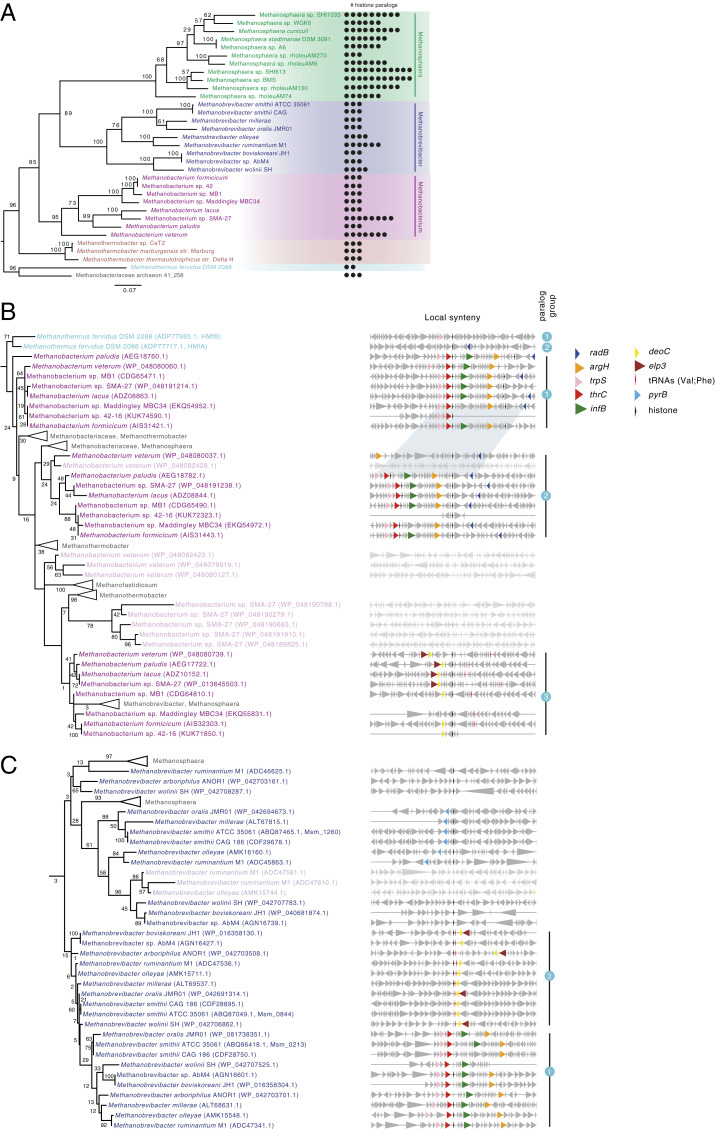
Phylogenetic analysis of histone paralogs in the Methanobacteriales. (*A*) Maximum-likelihood reference phylogeny (species tree) of the order Methanobacteriales, using IF-2a as a representative, vertically inherited gene. Bootstrap values are shown as a percentage out of 200 nonparametric bootstraps. The tree is rooted with *M. fervidus* and Methanobacteriaceae archaeon 41_258 as the outgroup. The number of histone paralogs in a given genome is mapped on the right-hand side. (*B* and *C*) Examples of recent duplications (lighter taxon labels) and long-term maintenance of paralogs in the genera *Methanobacterium* (*B*) and *Methanobrevibacter* (*C*), as supported by tree topology and conserved synteny. Note the clustering of proteins according to shared synteny rather than by species. Examples of paralog groups (1/2/3) are highlighted. Shared synteny across the *Methanobacterium*/*Methanobrevibacter* divide supports paralogous relationships (groups 1 and 3). Even deeper paralogy is suggested by the fact that local spatial association of *trpS* (group 1) and *radB* (group 2) with histones extends to *M. fervidus.* Longer gaps in the synteny blocks, evident for individual genomes, are the result of incomplete genome annotations. Genes are automatically color coded based on similarities in functional annotation (*Methods*). The trees are rooted with reference to a wider phylogeny of archaeal histones (*Methods*) and bootstrap values shown as a percentage out of 500 nonparametric bootstraps. In both *B* and *C* some sequences from other Methanobacteriales have been collapsed for clarity. The scale bar represents the average number of substitutions per site. Full trees and the underlying alignments are provided as Datasets S2–S7.

Phylogenetic analysis of histones across archaea is complicated by the fact that histones are short (<70 amino acids) and timescales are large, leading to poorly supported nodes in a global phylogeny of archaeal histones (*Methods* and Datasets S2–S7). We therefore focused our analysis on the Methanobacteriales, which include *Methanosphaera*, *Methanobrevibacter*, and *Methanobacterium* spp. as well as *M. fervidus* (Fig. 4*A*). Alongside abundant lineage-specific duplication events (lighter taxon labels in Fig. 4 *B* and *C*), we find several cases of longer-term paralog maintenance, indicated by the existence of multiple groups of sequences that each recapitulate the species phylogeny (Fig. 4 *B* and *C* and *SI Appendix*, Fig. S8). For example, branching patterns—as well as conserved synteny—of histones in *Methanobacterium* strongly suggest two ancient gene duplication events that preceded the divergence of this genus (Fig. 4*B*). Importantly, synteny analysis also reveals maintenance of paralogs between *Methanobrevibacter* and *Methanobacterium* (groups 1 and 3 in Fig. 4 *B* and *C*), indicating that these originated from even more ancient duplications, dating back to the ancestor of these two genera. As synteny breaks down further, making confident assignments becomes harder. Closer inspection of local gene neighborhoods, however, suggests that there might be even deeper conservation of recognizable paralogs all the way out to *M. fervidus*, where *hmfB* (*hmfA*) is flanked upstream (downstream) by *trpS* (*radB*), whose relative position is conserved in *Methanobrevibacter* and *Methanobacterium* spp. (Fig. 4 *B* and *C*). We found no evidence for gene conversion between paralogous histones in this clade (*Methods*).

To put the timescale of paralog origin into context, we note that the lineages leading to *M. stadtmanae* and *M. smithii* split an estimated ∼1.3 Gya, while the wider Methanobacteriales are thought to have emerged as a clade ∼1.6 Gya (21). At least some archaeal histone variants have therefore been maintained for hundreds of millions of years of evolution, rendering them comparable in age to the oldest known eukaryotic histone variants, which date back to the last common ancestor of eukaryotes, roughly 1.2 to 2 Gya (22, 23).

**Fig. 5.**
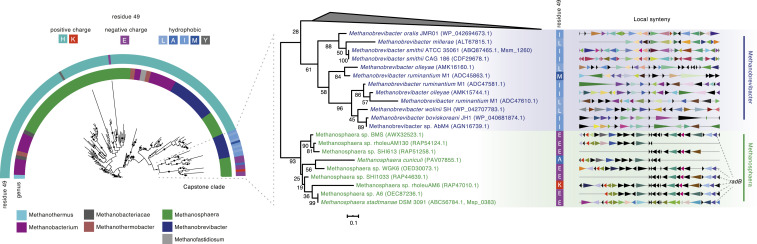
Phylogenetic analysis of capstone histones. A maximum-likelihood tree including all Methanobacteriales genomes (Dataset S7) is displayed at *Left*, along with information on amino acid identity at residue 49. Histone paralogs with capstone properties (negatively charged or hydrophobic amino acids) in *Methanosphaera* and *Methanobrevibacter* spp. cluster to the exclusion of other histones found in these species. Local synteny in the vicinity of histone paralogs is shown at *Right*. Genes are automatically color coded based on similarities in functional annotation. The *radB* gene is highlighted to allow cross-referencing with Fig. 4 *B* and *C*. Bootstrap values are shown as a percentage out of 500 nonparametric bootstraps. The scale bar represents the average number of substitutions per site.

Regarding capstones, we find evidence for shared vertical descent of the *M. stadtmanae* and *M. smithii* capstones (Fig. 5). At the same time, we note that histones with negatively charged/hydrophobic amino acids at residues 49 are also present in multiple independent lineages outside the Methanobacteriales, including members of the Hadesarchaea and Nanohaloarchaeota (*SI Appendix*, Fig. S7 and Table S1). Additional MD simulations for histones from each of these clades show unstable dimer:dimer interfaces like those we observed for *M. stadtmanae* and *M. smithii* capstones (*SI Appendix*, Fig. S7). In as much as amino acid identity at residue 49 can be used as a diagnostic marker, this suggests that capstone functionality has evolved multiple times independently.

### Single Amino Acid Changes Underpin Functional Differences between Paralogs.

The case of *M. stadtmanae* Msp_0383 illustrates that substitutions of individual amino acids can have strong effects on histone properties and, ultimately, chromatin state. This is also true in eukaryotes (1, 24, 25). H3.3 and H3.1, for example, differ in only four amino acids (three of which are located in the histone fold domain), but are recognized by different chaperones, deposited at defined locations along the genome, and make distinct, nonredundant contributions to genome function, notably during gametogenesis (20, 26, 27).

**Fig. 6.**
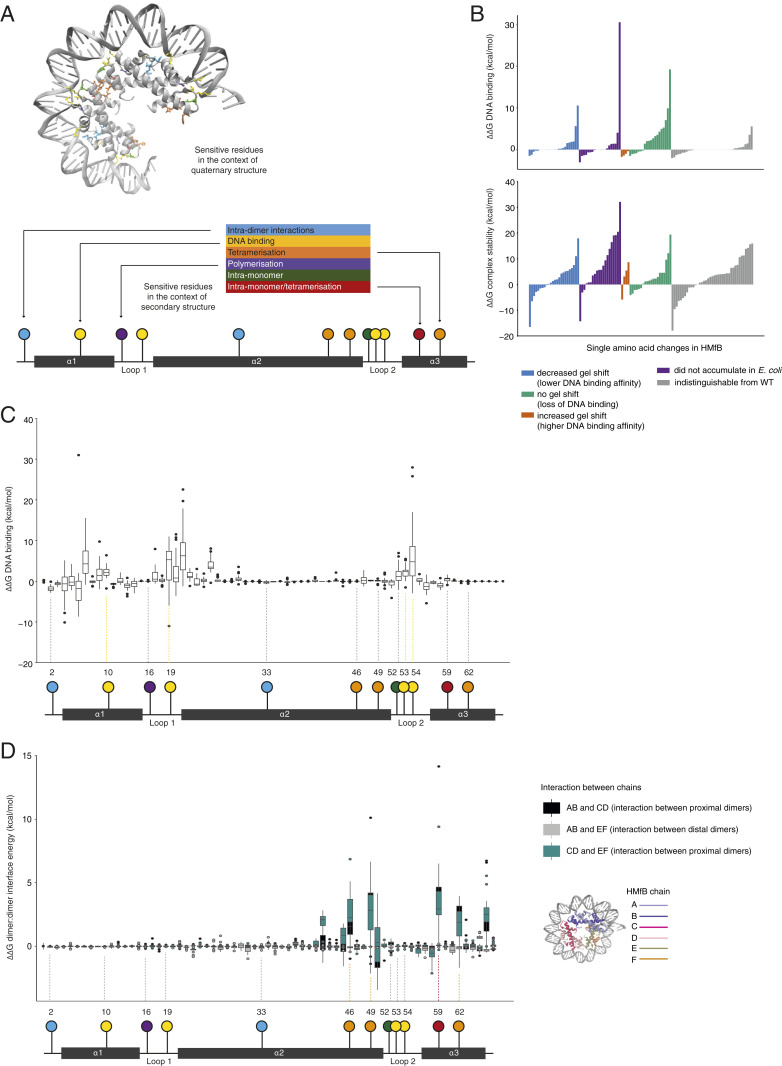
Modeling the impact of single amino acid mutations on the (HMfB)_6_–DNA complex. (*A*) Residues where mutations are known from previous experimental work (3, 18, 28–30) to affect monomer:monomer interactions, DNA binding, tetramerization, polymerization, and intramonomer interactions are highlighted on the quaternary and secondary structures. (*B*) FoldX-calculated changes in DNA binding affinity (*Top*) and stability (*Bottom*) for HMfB single amino acid mutants previously characterized qualitatively in gel shift experiments (30). Individual mutations are listed in *SI Appendix*, Table S2. (*C* and *D*) DNA binding (*C*) and tetramerization strength (*D*) for all possible single amino acid mutations of HMfB. The location of residues with previously known function is shown on the secondary structure beneath. For *D*, the resulting interaction energy between each dimer pair in the hexamer was calculated and the location of dimer pairs in the hexamer is shown. ΔΔG is quoted relative to the wild-type HMfB structure for all plots.

To understand how specific amino acid changes underpin the functional diversification of archaeal histone paralogs, we integrated structural modeling and evolutionary analysis. First, we used the FoldX forcefield (*Methods*) to in silico mutate each amino acid in the model histone HMfB from *M. fervidus* to every other possible amino acid to identify sites particularly sensitive to change. We then compared these predicted effects to previous in vitro work on HMfB, which had identified residues that, when mutated, affect DNA binding, the direction of DNA supercoiling, rigidity of the histone–DNA complex, thermostabilization, oligomer formation, and the ability of the histone to accumulate in *Escherichia coli*, a proxy for folding stability (3, 18, 28–30). We find that predicted and observed effects are highly concordant (Fig. 6 *A* and *B* and *SI Appendix*, Table S2). For example, our fast mutational scanning identifies the four residues (46/49/59/62; Fig. 6*D*) previously highlighted as critical for stable tetramerization (8, 18) and we predict, more often than not, whether, in previous gel shift assays (30), a specific mutation had led to increased (stronger DNA binding) or decreased (weaker DNA binding) mobility (Fig. 6*B*). This high degree of congruence provides additional validation for our modeling approach. It also increases our confidence in predictions of structural sensitivity for residues that have not been experimentally interrogated. For example, residues 21 and 50, for which no experimental data are available, show large deviations in DNA binding affinity and tetramerization strength, respectively, when mutated (Fig. 6*D*).

**Fig. 7.**
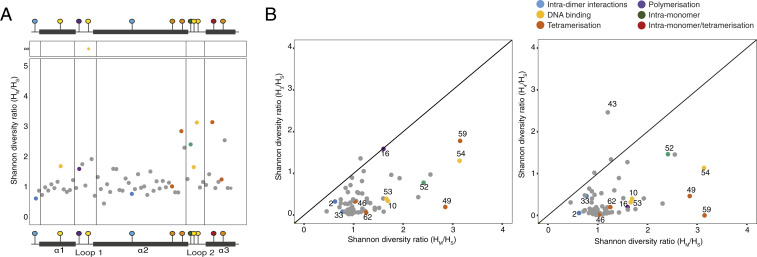
Comparative analysis of sequence diversity in archaeal and eukaryotic histones. (*A*) Shannon diversity ratio (*H*_*M*_*/H*_*S*_) at each position in the core histone fold domain. Residues are colored by key function from previous mutational studies (Fig. 6). *H*_*S*_ for residue 19 is 0, so the Shannon ratio is undefined. (*B*) Shannon diversity ratios for H3 (*H*_*3*_*/H*_*S*_, *Top*) and H4 (*H*_*4*_*/H*_*S*_, *Bottom*) compared to *H*_*M*_*/H*_*S*_. Residues of particular interest are numbered.

Next, we asked how this comprehensive landscape of possible effects compares with substitutions that actually occurred during the evolution of archaea. Do structurally sensitive sites remain largely conserved across paralogs? Or are changes at key sites, like those we observe for Msp_0383, relatively commonplace? To answer this question in a pan-archaeal manner, we took a nonphylogenetic approach. We aligned the 506 archaeal histone proteins in our sample (*Methods*) and then split them into two groups, depending on whether they come from a genome that encodes only a single histone gene or from a genome that encodes two or more paralogs. Our objective here was to identify residues along the histone fold that have become more diverse in multiparalog systems, where relaxed constraint or positive selection could drive diversification following duplication. Fig. 7 shows the amino acid diversity ratio *H*_*M*_/*H*_*s*_ for each residue, where *H*_*M*_ and *H*_*s*_ are Shannon diversity indexes calculated for a given residue (column in the alignment) across multihistone and single-histone genes, respectively (*Methods*). The average Shannon ratio will be affected by phylogenetic sampling, the number of histones in each group, and other factors and is therefore relatively uninformative. What is informative, however, are deviations from this average at specific residues.

Strikingly, diversification in species with multiple paralogs is strongly associated with structurally critical residues (Fig. 7*A*). This includes the capstone residue 49, but also several residues that make large contributions to DNA binding (10/19/53/54; Figs. 6 *C* and *D* and 7*A*), concentrated in the loop regions of the histone fold, and loop 2 in particular. Perhaps the most egregious example is residue 19 in loop 1, which is perfectly conserved as an arginine in single-histone archaea (*H*_*s*_ = 0) but accommodates eight different amino acids across the multihistone archaea in our sample. This suggests a significant change in the evolutionary regime at this site once more than two histones are present in the system. Given the strong deviation from the baseline diversity ratio, we think that positive selection is likely implicated in the diversification process rather than relaxed constraint alone.

Although we do not explore this extensively here, we note that residue-level diversification has some phylogenetic structure. Some residues exhibit a narrow phyletic pattern (*SI Appendix*, Fig. S9). Notably, this includes residue 54, which forms a conserved interaction with residue 19 (3). Diversification at this residue is confined almost entirely to the Asgard clade and excluding this clade from the analysis dramatically reduces diversity at residue 54 (*SI Appendix*, Fig. S10).

### Diversification of Eukaryotic versus Archaeal Histone Folds.

The residues involved in archaeal histone tetramerization are also important for interactions at the interface of two H3 molecules from neighboring H3:H4 dimers (31, 32). How, then, do archaeal histone paralogs compare to eukaryotic histone variants? Did diversification of the core histone fold follow a similar path? To address this question, we first added eukaryotic H3 and H4 sequences to our preexisting alignment of archaeal histones (*Methods* and *SI Appendix*, Table S3). We then calculated Shannon diversity indexes for H3 (*H*_*3*_) and H4 (*H*_*4*_) proteins found across eukaryotes and compared *H*_*4*_*/H*_*s*_ and *H*_*3*_*/H*_*s*_ to *H*_*M*_*/H*_*s*_. We find that diversification dynamics across the histone fold follow a similar pattern in multihistone genes and H3 (rho = 0.40, *P* = 0.0081) and to a lesser extent also H4 (rho = 0.24, *P* = 0.063). Residues 2 and 33, which are involved in intramonomer interactions, are not diverse in H3, H4, or archaeal histones. Substitutions at these positions may prevent the formation of the tertiary histone fold structure and are therefore selected against. Conversely, residues around the loop 2 region in particular experience accelerated diversification in both H3 and multihistone archaea relative to single-histone archaea. These similarities notwithstanding, several residues show conspicuous diversification in multiarchaeal histones but not H3/4, and vice versa. This includes residues 49 (high *H*_*M*_*/H*_*s*_, low *H*_*3/4*_*/H*_*s*_), 59 (high *H*_*M*_*/H*_*s*_, low *H*_*4*_*/H*_*s*_), and 43 (low *H*_*M*_*/H*_*s*_, high *H*_*4*_*/H*_*s*_). In addition, even residues with high diversity ratios in both eukaryotes and archaea only partially explore the same part of sequence space and tend to evolve toward different sets of amino acids (*SI Appendix*, Fig. S10). Our results therefore suggest that histone variants from archaea and eukaryotes independently focused their exploration of structural–functional space on structurally sensitive sites in the loop 2 region but also highlight significant lineage-specific constraints on histone evolvability.

## Discussion

Prior observations—from variable expression along the growth cycle to differential phenotypic effects upon deletion (11, 13, 14)—pointed to functional diversity of archaeal histone paralogs. The observations we report here not only reinforce this notion but also demonstrate that some histone paralogs in archaea have been maintained as distinct functional units over long evolutionary timescales, akin to eukaryotic histone variants.

Our modeling results suggest that paralogs, by exploiting the combinatorial opportunities of histone oligomerization, can generate diverse chromatin states at the level of individual histone–DNA complexes and enable both subtle, graded dosage-driven transitions and more radical changes such as those associated with the expression of capstones. We explored one of these transitions in *M. stadtmanae*, where the relative expression of different histone paralogs changes in stationary versus exponential phase. Based on our structural modeling and empirical protein abundance data, we predict that stationary phase in *M. stadtmanae* (as well as *M. smithii*) should be characterized by a larger fraction of less stable histone–DNA complexes. This is, arguably, unexpected given opposite trends inferred for *M. fervidus* and other hyperthermophiles and the general notion that stationary phase is associated with greater chromatin compaction. Experimental data will ultimately be required to determine whether this inferred difference is genuine or not. However, the discrepancy serves as a timely reminder to highlight the limitations of our modeling approach, which does not consider absolute histone titers, changes in intracellular conditions (e.g., in terms of solutes), and expression of other abundant architectural proteins (e.g., Alba) that will codetermine higher-order chromatin states. In this regard, our results should be considered a valuable starting point and incentive for further exploration rather than the final word, hewn in stone, on comparative chromatin complexity in archaea. Substantial further work, both in vitro and in vivo, will be required to elucidate why certain histone properties have been selected for in different lineages and how individual paralogs are deployed in physiological context.

It is also worth noting that we examined only a small branch of the archaeal tree in depth, did not consider archaeal histone with tails or large indels (8, 33), and did not explore interactions and combinatorial complexity beyond the tetramer level. Our estimates of archaeal capacity to generate different chromatin states are therefore likely conservative. In particular, tetramer models do not allow us to consider stacking interactions between nonadjacent dimers, which affect oligomerization propensity (3, 8). Substantial additional complexity might further emerge from the consideration of N-terminal tails, which are present in some Heimdallarchaea (3, 8), the closest known relatives of eukaryotes (16). Studying these archaea and their tails will be particularly important to understand what—in the context of histone-based chromatin—constitutes eukaryotic innovation, elaboration, or shared archaeal heritage. This includes the question of whether “deep paralogy” might exist between extant eukaryotic and archaeal histones, something our results do not directly imply.

Based on our current knowledge, we speculate that paralog-mediated structural change might play an outsize role in archaea compared to eukaryotes, where posttranslational modifications and interactions with other proteins are heavily involved in altering chromatin state in response to upstream signals. One of the key eukaryotic innovations might have been a switch from predominantly paralog-based generation of different chromatin states to using an octameric nucleosome as a platform for integrating epigenetic information. This innovation might also have enabled another: local specification. In eukaryotes, divergent regulatory states can be encoded along the same chromosome via targeted deposition of paralogs and histone marks by enzymes and chaperones that can interact with specific histones, DNA sequences, and/or other constituents of chromatin. At present, we have no evidence that the capacity for such local control exists in archaea. Current data support only a global, genome-wide role in reshaping chromatin state. It will be interesting in the future to determine whether complexes of different composition are indeed randomly distributed or show nonrandom patterns along archaeal chromosomes in a manner anticipating eukaryotic chromatin. To this end, we need to develop a better understanding of archaeal histone variants in physiological context. The specific functional roles of archaeal variants in the context of genome function remain entirely unknown, a glaring gap that can only be plugged by in vivo experiments. Our study provides ample incentive for further research to establish how archaeal paralogs are regulated, how they interact with other DNA-binding proteins to determine global and perhaps local chromatin states, and how paralogs contribute to adaptive responses in physiological context.

## Methods

### Alignment of Histones.

A previously compiled set of archaeal histone proteins (10) was filtered to include only proteins between 60 and 80 amino acids in length with a single histone fold (*SI Appendix*, Fig. S11). For reference, HMfB is 69 amino acids long. Sequences were further filtered to randomly remove redundant entries (i.e., sequences 100% identical to another entry). This filtered set of histones from 282 species of archaea (139 with more than one histone, 143 with one histone) was aligned using MAFFT-linsi (-localpair -maxiterate 1000) (34). Eukaryotic H3 and H4 protein sequences were downloaded from InterPro (matching folds IPR007125, IPR035425, and IPR032454) (35), filtered for length (95 to 110 amino acids for H4, 130 to 145 amino acids for H3; *SI Appendix*, Fig. S11), and added to the archaeal histone alignment using MAFFT-linsi (-localpair -maxiterate 1000 -seed) after removing H2A/B sequences. Positions where more than 5% of sequences had a gap were removed from further analysis.

### HMfB Single Mutants.

We used the BuildModel command in FoldX (36) to introduce all possible single amino acid changes into the HMfB hexamer (Protein Data Bank [PDB] structure 5T5K). All six histone monomers in the structure were mutated simultaneously. FoldX refines structures by minimizing the energy of mutated side-chain residues and neighboring residues according to its empirically derived forcefield. The positions of nonadjacent residues and all peptide backbone atoms remain fixed. Although MD simulations are more rigorous to determine accurate binding affinities and allow us to sample the dynamics of the complex (below), FoldX allows us to sample, at high throughput, changes in energy associated with mutations at individual positions in the protein. We therefore refer to this approach as a fast mutational scanning technique. FoldX was used at the default temperature setting of 298 K. We calculated the relative change in Gibbs free energy (∆∆G) of the system, DNA binding, and tetramerization energies for each mutant using FoldX relative to the minimized HMfB hexamer structure.

The Gibbs free energy (Eq. **1**) is a thermodynamic quantity defined as the amount of reversible work a mechanical system can undergo, where ∆H is the enthalpic contribution and ∆S is the entropic contribution. By calculating the sum total of inter- and intramolecular forces, determined by the FoldX forcefield, we can calculate ∆G and predict the structural stability of the complex. By subtracting ∆G_mutant_ from ∆G_wildtype_ of HMfB we arrive at the relative change in Gibbs free energy, ∆∆G (Eq. **2**). The binding affinity can be determined by subtracting the energetic contribution from the DNA and histone from the complex (Eq. **3**). The same can be said for the histone tetramerization energy; subtracting dimer energies from the tetramer energy will leave us with the energetic contribution of tetramerization:

ΔG=ΔH−TΔS [1]

$$\Delta \Delta \text{G}=\Delta {\text{G}}_{\text{mutant}}-\Delta {\text{G}}_{\text{wildtype}}$$ [2]

$$\Delta {\text{G}}_{\text{bind}}=\Delta {\text{G}}_{\text{complex}}-\Delta {\text{G}}_{\text{DNA}}-\Delta {\text{G}}_{\text{histone}}.$$ [3]

### Tetramer Models of Archaeal Histones.

The HMfB tetramer model was built by removing one histone dimer (chains E and F) and 30 bp of DNA from the 5T5K PDB structure. For each species, all possible combinations of histone monomers were modeled as a tetramer, with the following exceptions: To enable fair structural comparison, we analyzed only models where no histone carried a deletion in the core histone fold (HMfB residues 2 to 65) and considered only histones 60 to 80 amino acids in length. We focused on tetramers as this allows DNA binding and tetramerization strength to be calculated without assuming that histones assemble into longer oligomers. Substitutions at positions in the core histone fold were mapped onto the HMfB tetramer using the BuildModel function of FoldX (36). Structures were energy minimized for 10,000 steps of combined steepest descent and conjugate gradient using AmberTools. Unlike FoldX, which minimizes only mutated side-chain residues and their neighbors, we used an all-atom minimization (using AMBER ff14SB) but avoided any significant refolding by applying a 2-kcal⋅mol^−1^⋅Å^−2^ harmonic restraint on backbone atoms.

Binding affinity and tetramerization energies were calculated using the single-trajectory molecular mechanics Poisson–Boltzmann surface area (MMPBSA) approach (37). In this method we decompose ∆H in Eq. **1** into the gas phase energy and the free energy of solvation (Eq. **4**). The gas phase energy was calculated as the total of energy from the AMBER ff14SB forcefield (38) and the free energy of solvation was approximated using the Poisson–Boltzmann equations:

$$\Delta \text{G}=\left({\text{E}}_{\text{gas}}+{\text{E}}_{\text{solv}.}\right)-\text{T}\Delta \text{S}.$$ [4]

In our present energy minimization scheme, single mutations will not significantly change the conformation of the histone and the relative change in entropy, ∆∆S, will be close to zero. For this reason, we have not included the entropic contribution to the Gibbs free energy values. This is not to say that a single-residue mutation will never perturb the conformational landscape to an extent that would lead to a significant change in the entropic contribution to energy. Although rare, these processes may happen over longer timescales that are not accessible using our current methods.

∆∆G was calculated relative to the Msp_0769 homotetramer for *M. stadtmanae* tetramer models and relative to the HMfB homotetramer in all other cases.

### MD Simulations of *M. stadtmanae*.

Complexes of homotetrameric histones with DNA were parameterized using the Amber ff14SB potentials for canonical proteins using tLeap in AmberTools. Residues present in the sequence but removed in the filtering stage after alignment were manually added to the “full model” homotetrameric structures generated by FoldX and the complexes were energy minimized as above. Models were solvated with 14 Å of transferable intermolecular potential with 3 points water and neutralized with NaCl (∼0.18 M), countering the overall negative charge of the DNA backbone. Energy minimization was performed for 2,000 steps using combined steepest-descent and conjugate gradient methods. Following minimization, 20 ps of classical molecular dynamics (cMD) was performed in the constant temperature, constant volume (NVT) ensemble using a Langevin thermostat (39) to regulate the temperature as we heated it up from 0 to 300 K. Following the heat-up phase, we performed 100 ns of cMD in the isobaric/isothermal (constant temperature, constant pressure [NPT]) ensemble using the Berendsen barostat (40) to maintain constant pressure of 1 atm during the simulation. All simulations were performed using GPU (CUDA) version 18.0.0 of PMEMD (41–43) with long-range electrostatic forces treated with particle-mesh Ewald summation (44). MMPBSA calculations for DNA binding affinity and tetramerization strength were performed from frames 1,500—where the rmsd of each trajectory had started to equilibrate—to the end.

### Phylogenetic and Evolutionary Analysis.

To build an initial tree of archaeal histones, we queried all 282 species present in the structural analysis and available through NCBI with hmmsearch (HMMer suite, http://hmmer.org) and considered all single-domain hits against Pfam model CBFD_NFYD_HMF (PF00808, Pfam v.23) that were filtered out from the initial dataset. For reproducibility purposes, the Pfam gathering threshold was used as the thresholding option of hmmsearch (–cut_ga). Sequences were first aligned with MAFFT-linsi (using blosum30) and an initial tree inferred with IQ-TREE2 (automatic substitution model estimation: LG+R6 substitution model, 1,000 ultrafast bootstraps) (45). We then considered the minimal subtree containing all histones from *M. stadtmanae*. To extend the diversity of Methanosphaera histones, we downloaded additional available Methanosphaera genomes from the NCBI refseq database. All sequences were then realigned using MAFFT-linsi (using blosum62), and a maximum-likelihood tree was built using RAxML-ng (500 nonparametric bootstraps, LG substitution model) (46). Trees were visualized using iTol (47) and local synteny using Genespy (48). A reference species tree was built using RAxML-ng (LG substitution model, 200 bootstraps) based on a MAFFT-linsi alignment of IF-2a proteins (identified as hits against the TIGR00491 HMM model). This tree recapitulates previously inferred relationships among the Methanobacteriales (49).

To establish whether branching patterns might be confounded by gene conversion, we searched an alignment of all Methanobacteriales histones for signals of recombination/gene conversion using GARD (50), PHIpack (51), and RDP4 (52). GARD and PHIpack were run with standard settings using 1,000 permutations and a window size of 100 bp for PHIpack. RDP4 was also run with default settings using all available methods (RDP, GENECONV, Chimaera, MaxChi, 3Seq, BootScan, and SiScan). BootScan and SiScan primary scans were included. A window size of 30 was used for RDP. For MaxChi and Chimaera, the number of variable sites per window was 70 and 60, respectively. Not a single gene conversion event was supported by GARD or PHIpack or the RDP4 consensus. Tentative events called by individual methods as part of the RDP4 pipeline were investigated manually and confirmed not to affect phylogenetic inference. Overall, we found very little support for the hypothesis that gene conversion has played a major role during evolution of histones in this clade, consistent with the observed clustering of paralogs in a manner that recapitulates species phylogeny.

Diversity at a given residue (column in the alignment) and for a given group (e.g., archaea with multiple histone paralogs) was calculated using the Shannon diversity index (*H*). Subsequently, we computed diversity ratios for two groups (A and B) as

$$Shannon\;diversity\;ratio=\;\frac{Shannon\;diversity\;index\;for\;A}{Shannon\;diversity\;index\;for\;B}.$$

Similarity between histone groups in terms of the types of amino acids found at a given residue was calculated using the Jaccard index formula.

### Histone Expression Levels for Different Species.

For *M. smithii*, *T. kodakarensis*, and *Thermococcus onnurineus*, histone mRNA levels in exponential phase were obtained from NCBI’s Gene Expression Omnibus (GEO) and primary publications. The relative expression of histones in *T. kodakarensis* (53) and *T. onnurineus* (GSE85760) (54) is plotted as base mean and normalized mRNA, respectively, in *SI Appendix*, Fig. S4. For *M. smithii*, we used the median value of histone expression across all replicates and conditions for strain MsmPS as determined in ref. 55. Expression levels for *Thermococcus litoralis*, *Methanothermobacter thermoautotrophicus*, *Methanothermobacter marburgensis*, *Ferroglobus placidus*, *Archaeoglobus fulgidus*, and *Archaeoglobus profundus* were taken from comparative quantitative proteomics data reported in ref. 56. We did not include archaea from the latter study where, due to high sequence identity among paralogs, intensities could not be uniquely assigned to a single paralog (*Methanococcus jannaschii*, *Pyrococcus furiosus*).

### *M. stadtmanae* Culture, qRT-PCR Analysis, and Proteomics.

*M. stadtmanae* DSM3091 was grown as previously described (57). Briefly, cultures were grown at 37 °C in 50 mL minimal medium under strict anaerobic conditions. Medium was reduced with Na_2_S and cysteine (2 mM) and supplemented with 100 μg/mL ampicillin to prevent bacterial contamination. A 150-mM concentration of methanol and 1.5 atm H_2_-CO_2_ (80/20 [vol/vol]) served as carbon and energy source. Growth was monitored via turbidity at 600 nm (T_600_) and stopped at exponential or stationary phase by short incubation on ice (15 min) and subsequent centrifugation of cultures (3,200 × *g* for 30 min at 4 °C). Resulting cell pellets were resuspended either in 500 µL 50 mM Tris containing RiboLock (Thermo Fisher Scientific) for RNA isolation or in 500 µL 50 mM triethylammonium bicarbonate buffer for proteomics until further processing.

For both isolation of RNA and proteins, *M. stadtmanae* cells were lysed in liquid nitrogen using a Mikro-Dismembrator S laboratory ball mill (Sartorius) for 3 min at 1,600 bpm.

For proteome analysis, cells were centrifuged after homogenization at 15,700 × *g* and 4 °C for 30 min and supernatant was used as cell-free protein extracts. RNA extraction and qRT‐PCR assays were then performed as described earlier (58). mRNA expression levels of three biological replicates were calculated using the normalizing 2‐ΔΔCt value. *Msp_16S* and *Msp_rpoB* were used as genes for normalization (59). Primers used are provided in *SI Appendix*, Table S4.

Cell-free protein extracts were run on a gel, low-molecular-weight section (<10 kDa) excised, and processed using a procedure adapted from ref. 60. Briefly, excised gel sections were further cut into cubes of ∼2 × 2 mm and washed with 50 mM ammonium bicarbonate in 50% aqueous acetonitrile (ACN). Dehydration of gel sections was carried out with 100% ACN. Sections were then sequentially reduced and alkylated with 10 mM dithiothreitol and 55 mM iodoacetamide, respectively. Digestions were carried out by addition of 500 ng of trypsin per gel section, followed by incubation at 37 °C overnight. Gel digest supernatants were then dried completely by vacuum centrifugation. Following extraction of tryptic peptides from gel pieces, dried extracts were reconstituted in 1% aqueous ACN, 0.1% formic acid (FA). Desalting was performed using C18 reverse-phase solid-phase extraction spin tips (Glygen Corp.) following the manufacturer’s recommendations and eluted tryptic peptides were then dried by vacuum centrifugation.

Desalted gel digests were solubilized in 20 µL of 0.1% aqueous trifluoroacetic acid (TFA) and clarified solutions transferred to autosampler vials for liquid chromatography–mass spectrometry analysis. Peptides were separated using an Ultimate 3000 RSLC nanoliquid chromatography system (Thermo Scientific) coupled to a LTQ Velos Orbitrap mass spectrometer (Thermo Scientific) via an EASY-Spray source. Sample aliquots (5.0 μL per injection) were loaded in technical duplicate onto a trapping column (Acclaim PepMap 100 C18, 100 μm × 2 cm) at 8 μL/min in 2% ACN, 0.1% TFA. Peptides were then eluted online to an analytical column (EASY-Spray PepMap C18, 75 μm × 25 cm) and peptides were separated using a stepped 90-min gradient: 4 to 25% buffer B for 60 min, 25 to 45% buffer B for 30 min. Buffer compositions were buffer A, 2% ACN, 0.1% FA; buffer B, 80% ACN, 0.1% FA. Eluted peptides were analyzed by the LTQ Velos operating in positive ion polarity using a data-dependent acquisition mode. Ions were selected for fragmentation from an initial MS1 survey scan at 15,000 resolution (at *m/z* 200), followed by Ion Trap collisional induced dissociation (CID) of the top 10 most abundant ions. MS1 and MS2 scan automatic gain control (AGC) targets were set to 1e6 and 1e4 for maximum injection times of 500 and 100 ms, respectively. A survey scan with *m/z* range of 350 to 1,500 was used, with a normalized collision energy (NCE) set to 35%, charge state rejection enabled for +1 ions, and a minimum threshold for triggering fragmentation of 500 counts.

The resulting data were processed using the MaxQuant software platform (v1.5.3.8), with database searches carried out by the in-built Andromeda search engine against the *M. stadtmanae* DSM3091 proteome as annotated in NCBI. A reverse decoy database search approach was used at a 1% false discovery rate (FDR) for peptide spectrum matches and protein identifications. Search parameters included maximum missed cleavages set to 2, fixed modification of cysteine carbamidomethylation and variable modifications of methionine oxidation, protein N-terminal and lysine acetylation, glutamine to pyro-glutamate conversion, and asparagine deamidation as well as lysine and arginine methylation.

Label-free quantification (LFQ) was enabled with a minimum ratio count of 2. The “match between runs” function was used with match and alignment time settings of 0.7 and 20 min, respectively.

## Supplementary Material

Supplementary File

Supplementary File

Supplementary File

Supplementary File

Supplementary File

Supplementary File

Supplementary File

Supplementary File

Supplementary File

Supplementary File

## Data Availability

All study data are included in this article and *SI Appendix*.
