# Supplementary material for: Histone variants in archaea and the evolution of combinatorial chromatin complexity
Source: Proc Natl Acad Sci U S A. 2020 Dec 7;117(52):33384–95. doi: 10.1073/pnas.2007056117 (PMC7776873; doi:10.1073/pnas.2007056117)
Supplement: Supplementary File [file pnas.2007056117.sapp.pdf]

SUPPLEMENTARY APPENDIX

Included in this documents:

Figures S1-11
Tables S1-4
References

Provided outside of this document:

SI Movie 1. Video of molecular dynamics simulation trace for the Msp_0383 tetramer.

SI Movie 2. Video of molecular dynamics simulation trace for the Msp_0769 tetramer.

Dataset S1 (Dataset). Tetramerization and DNA binding energies for all homo- an heterotetrameric histone-DNA complexes in 282 archaea.

Dataset S2. Alignment of IF-2A orthologs (.fa format).

Dataset S3. Alignment of 560 archaeal histones (.fa format).

Dataset S4. Alignment of augmented set of 168 Methanobacteriales histones (including additional *M. stadtmanae* genomes) (.fa format).

Dataset S5. Reference phylogeny based on alignment in Dataset S2. Bootstrap values are given as a percentage of 200 non-parametric bootstraps (.nwk format).

Dataset S6. Pan-archaeal phylogeny of histones, reconstructed based on alignment in Dataset S3. Bootstrap values are given as a percentage of 1000 ultra-fast bootstraps (.nwk format).

Dataset S7. Phylogeny of histones in the Methanobacteriales, reconstructed based on alignment in Dataset S4. Bootstrap values are given as a percentage of 500 non-parametric bootstraps (.nwk format).

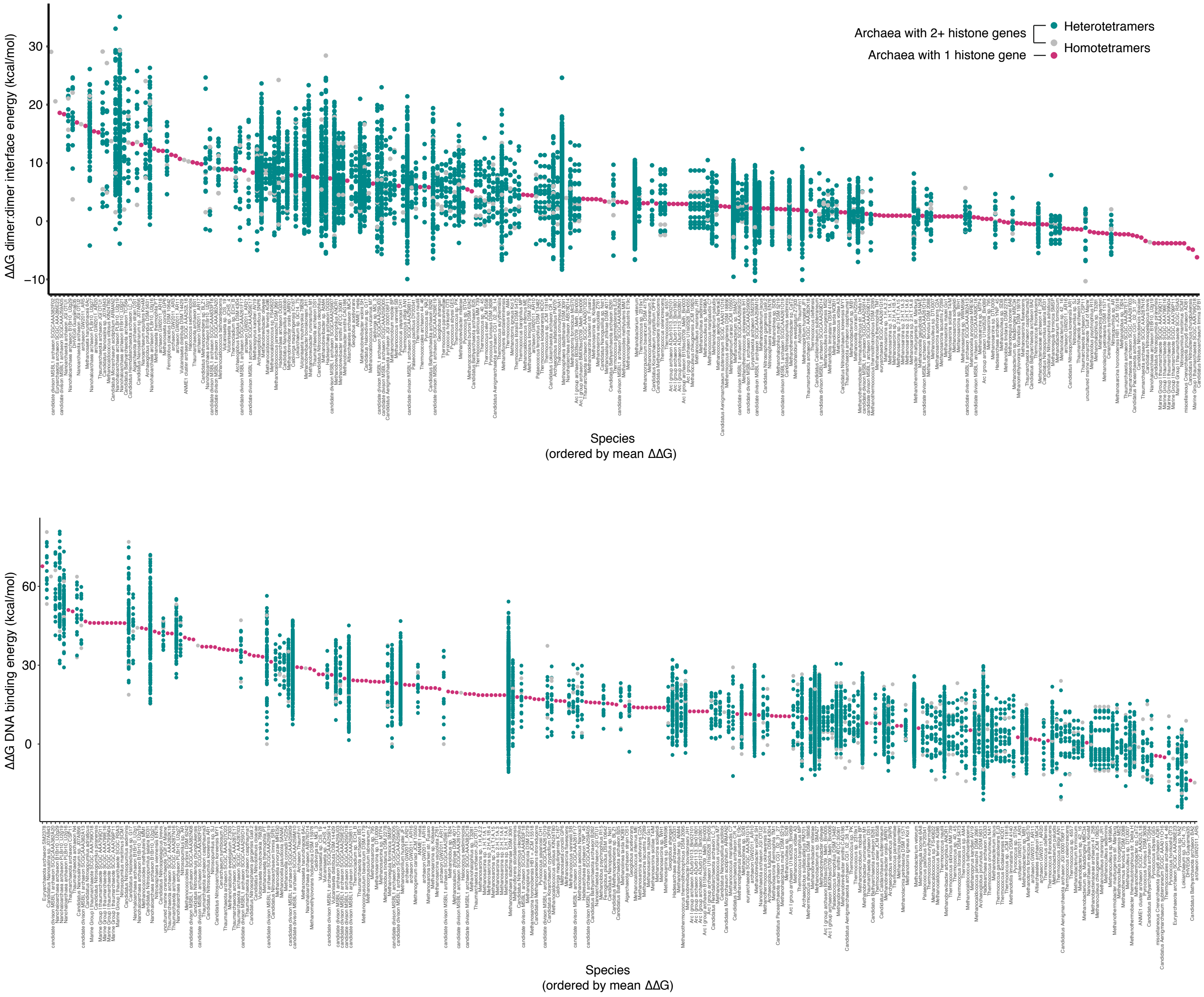

Figure S1. DNA binding strength and tetramerization strength (dimer:dimer interface energy) for every possible tetrameric histone complex within each species of archaea in our sample. Each point, grouped by species, represents an individual complex. Species are ordered by mean interaction energy across tetramers. ΔΔG is relative to HMfB.

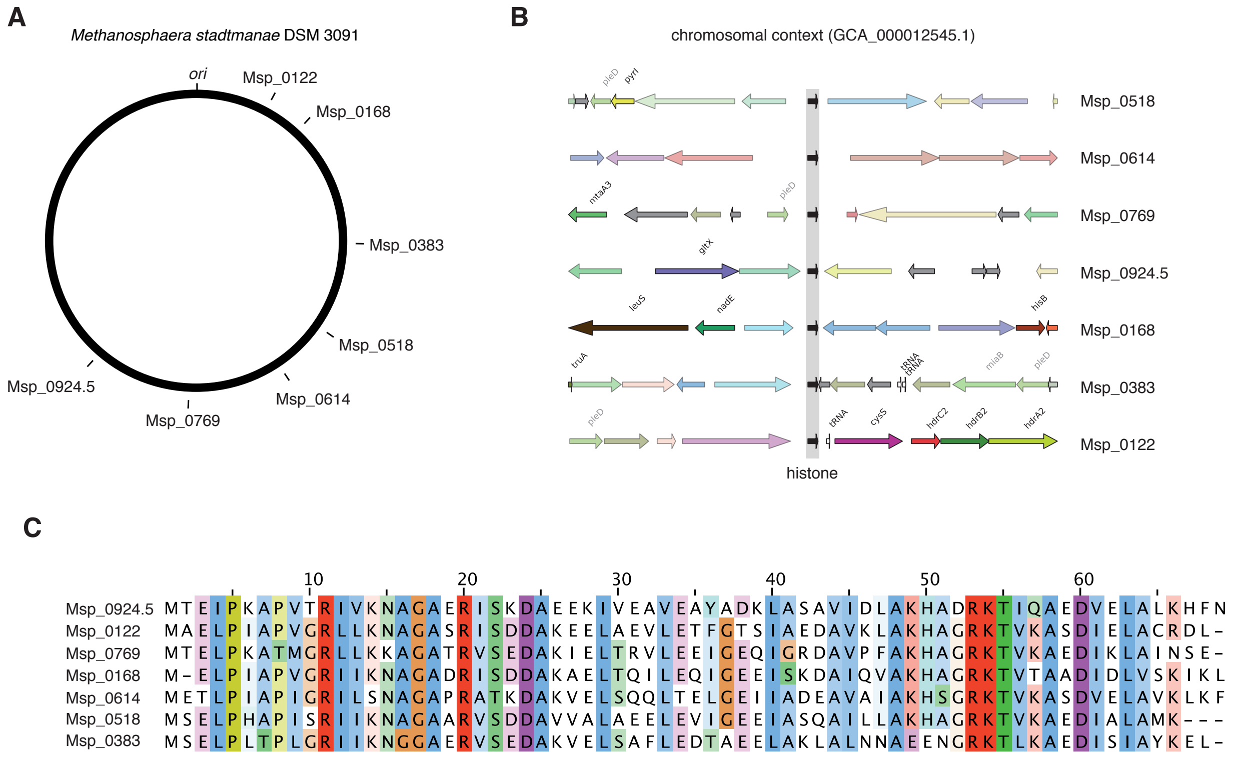

Figure S2. **(A)** Circular chromosome map of *M. stadtmanae* DSM 3091 showing the locations of the seven histone paralogs. Note that one of the histone paralogs is not annotated in GenBank. As it is located between Msp_0924 and Msp_0925, we have labelled it Msp_0924.5. **(B)** Chromosomal context of *M. stadtmanae* histone paralogs. Histones are flanked by comparatively large intergenic regions upstream and either large intergenic regions or convergently transcribed genes downstream, suggesting that histones are transcribed as single-gene operons. **(C)** Protein-level alignment of the seven paralogs.

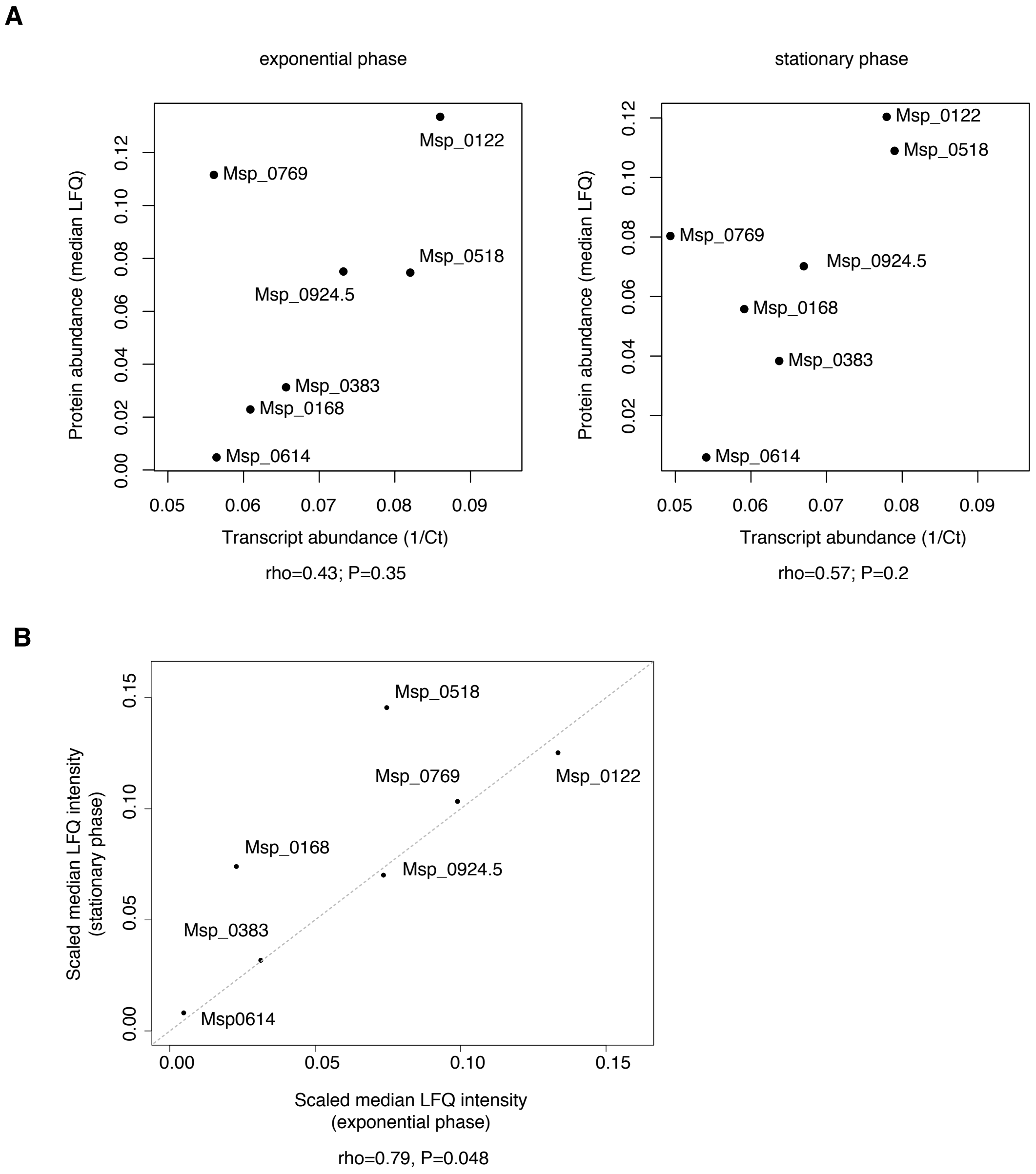

Figure S3. (A) Protein abundance and transcript abundance for *M. stadtmanae* histone paralogs in exponential (left) and stationary (right) phase. **(B)** Correlation between median protein abundance (LFQ intensity) for *M. stadtmanae* histone paralogs in exponential versus stationary phase.

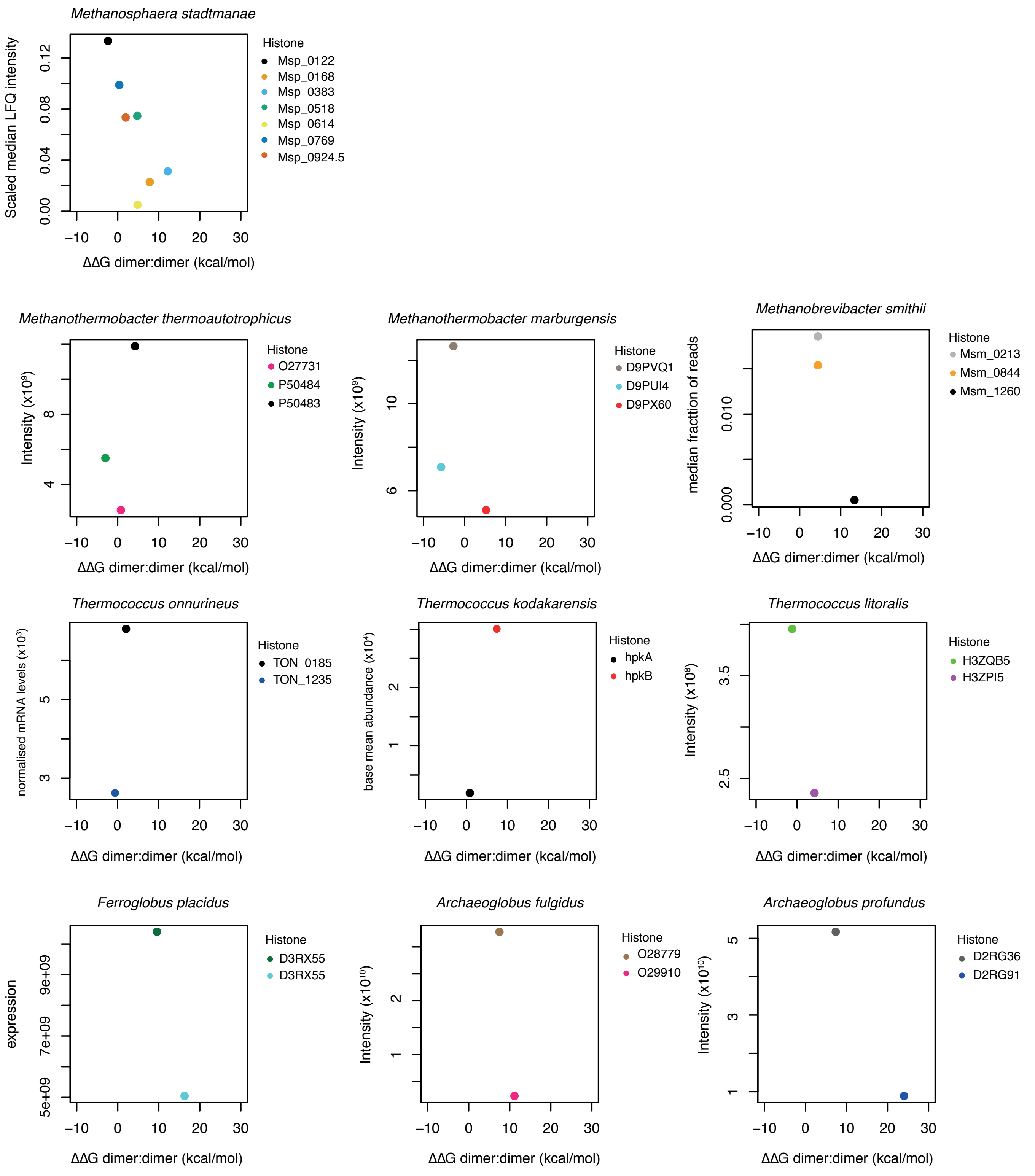

Figure S4. Tetramerisation strength for homotetrameric histone models in various archaea and its relation to paralog expression levels as measured by protein or transcript abundance in exponential phase. ΔΔG is relative to HMfB. See Methods for data provenance.

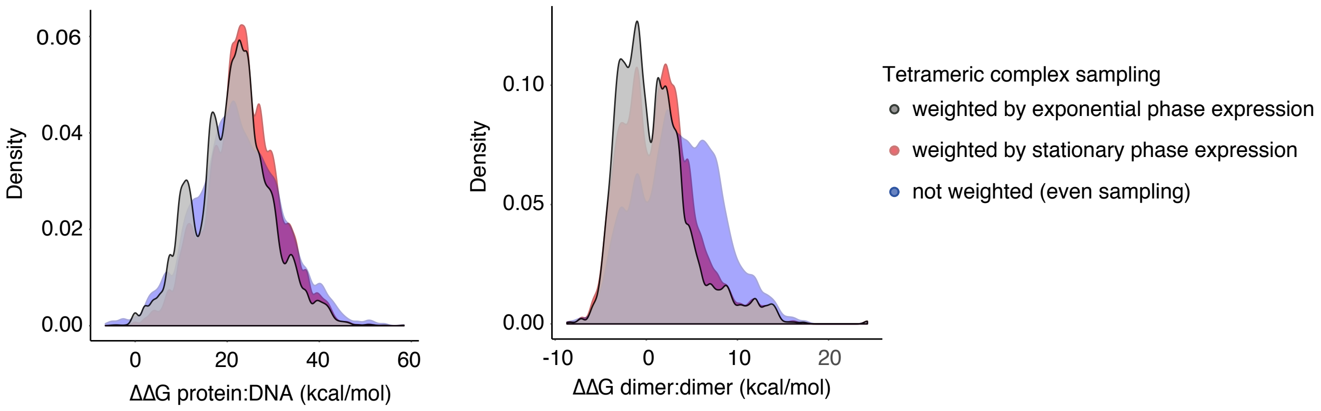

Figure S5. Distribution of DNA binding and tetramerization strength for 100,000 histone tetrameric complexes in *M. stadtmanae*, with tetramer composition determined by sampling based on relative paralog abundance (mean LFQ intensity) in exponential phase, stationary phase, and random sampling.

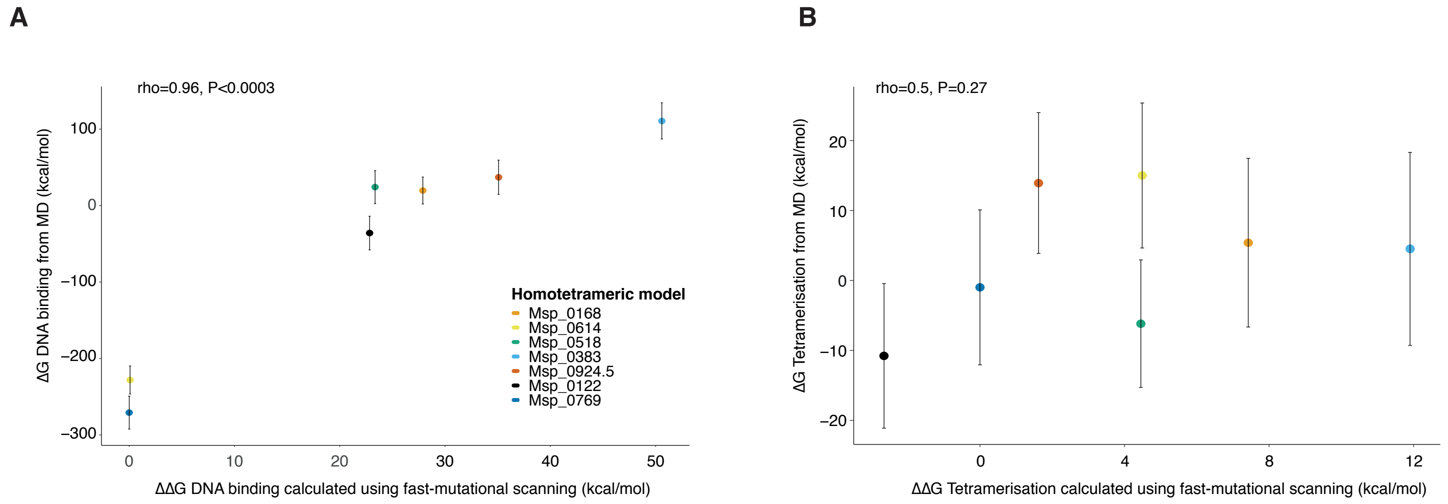

Figure S6. (A) DNA binding and **(B)** tetramerization energies across 100 ns of MD simulations compared to fast mutational scanning results (ΔΔG relative to Msp_0769) for homotetrameric histone complexes from *M. stadtmanae*. Tetramerization energies from MD are calculated for frames 1500-5000, i.e. following stabilization of RMSD.

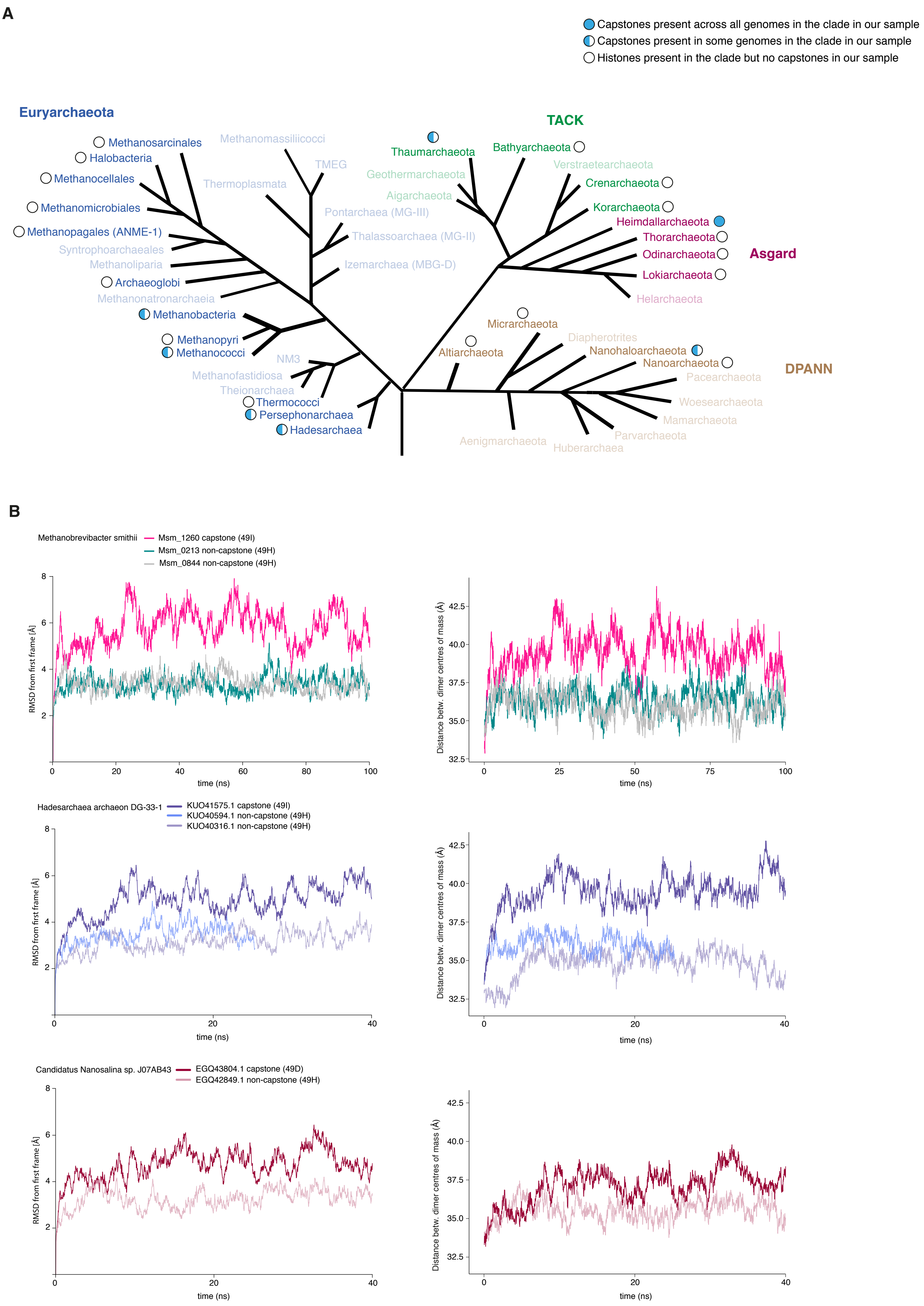
A
Capstones present across all genomes in the clade in our sample
Capstones present in some genomes in the clade in our sample
Histones present in the clade but no capstones in our sample
Euryarchaeota
Methanosarcinales
Halobacteria
Methanocellales
Methanomicrobiales
Methanopagales (ANME-1)
Syntrophoarchaeales
Methanoliparia
Archaeoglobi
Methanonatronarchaeia
Methanobacteria
Methanopyri
Methanococci
Methanomassiliicocci
TMEG
Thermoplasmata
Pontarchaea (MG-III)
Thalassoarchaea (MG-II)
Izemarchaea (MBG-D)
NM3
Methanofastidiosa
Theionarchaea
Thermococci
Persephonarchaea
Hadesarchaea
TACK
Thaumarchaeota
Bathyarchaeota
Geothermarchaeota
Aigarchaeota
Verstraetearchaeota
Crenarchaeota
Korarchaeota
Asgard
Heimdallarchaeota
Thorarchaeota
Odinarchaeota
Lokiarchaeota
Helarchaeota
DPANN
Micrarchaeota
Altiarchaeota
Diapherotrites
Nanohaloarchaeota
Nanoarchaeota
Pacearchaeota
Woesearchaeota
Mamarchaeota
Parvarchaeota
Aenigmarchaeota
Huberarchaea
B
Methanobrevibacter smithii
Msm_1260 capstone (49I)
Msm_0213 non-capstone (49H)
Msm_0844 non-capstone (49H)
RMSD from first frame [Å]
time (ns)
Distance betw. dimer centres of mass (Å)
Hadesarchaea archaeon DG-33-1
KUO41575.1 capstone (49I)
KUO40594.1 non-capstone (49H)
KUO40316.1 non-capstone (49H)
Candidatus Nanosalina sp. J07AB43
EGQ43804.1 capstone (49D)
EGQ42849.1 non-capstone (49H)

Figure S7. (A) Phyletic distribution of capstone candidates across archaea. Lighter shade of clade labels indicates that no histone sequence from a member of this clade is present in our sample. This can be either because no genome in that clade encodes histone proteins (e.g. Thermoplasmatales), or because no genomes from that clade were present in the initial analysis (e.g. Huberarchaea) or both. The tree is modified from *(1)*. **(B)** MD simulations of homotetrameric histone-DNA complexes for paralogs from *M. smithii* ATCC 35061, Hadesarchaea archaeon DG-33-1, and Candidatus Nanosalina sp. J07AB43 showing (left) RMSD and (right) distance between centre of mass of each dimer over up to 100 ns.

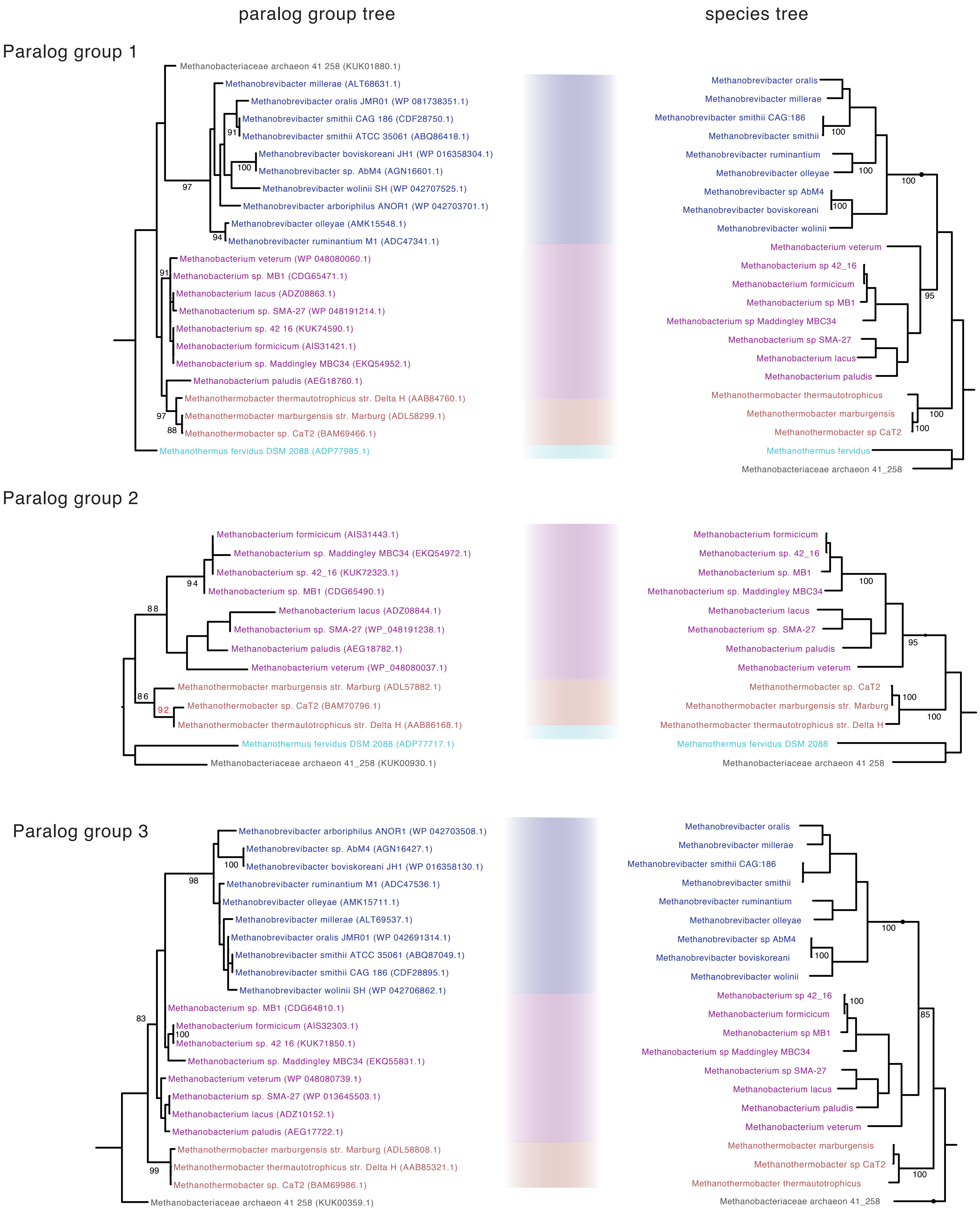

Figure S8. Trees representing individual paralog groups (see Figure 4B,C) recapitulate the species tree (see Figure 4A). Paralog group trees were extracted as subtrees from the larger Methanobacteriales histone tree (see Figure 4). As histone proteins are short and the phylogenetic signal in individual gene trees is limited, comparing each and every single branch between gene and species tree, including those with low support, is unlikely to be illuminating. To provide a robust and informative comparison, we therefore consider branches that – in a given paralog group tree – have high support (≥80), indicative of robust relationships that can be compared to the species tree. All but one high-support bifurcation in the paralog group trees recapitulate bifurcations observed in the species tree. The exception is highlighted in red (paralog group 2).

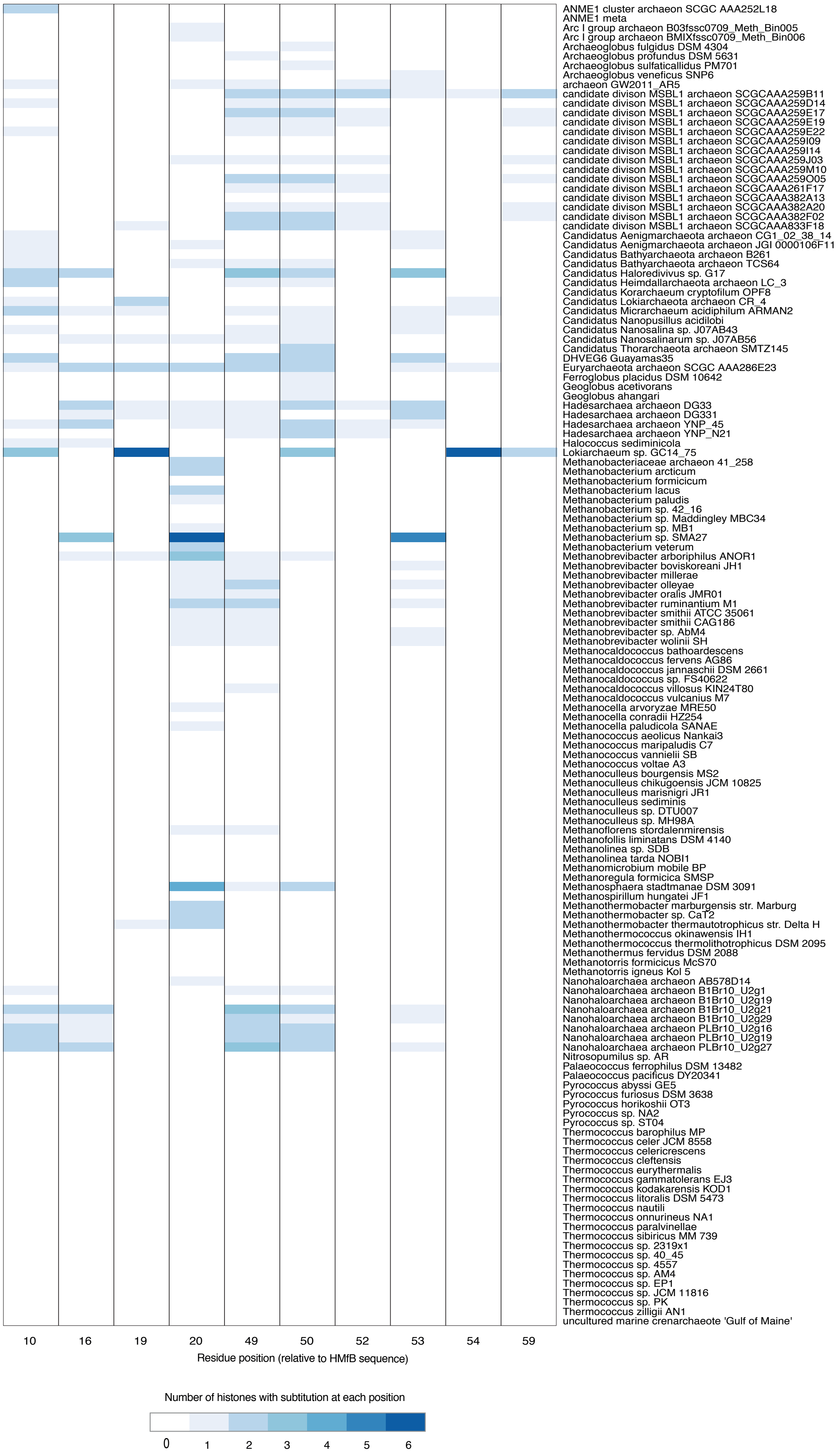
ANME1 cluster archaeon SCGC AAA252L18
ANME1 meta
Arc I group archaeon B03fssc0709_Meth_Bin005
Arc I group archaeon BMIXfssc0709_Meth_Bin006
Archaeoglobus fulgidus DSM 4304
Archaeoglobus profundus DSM 5631
Archaeoglobus sulfaticallidus PM701
Archaeoglobus veneficus SNP6
archaeon GW2011_AR5
candidate divison MSBL1 archaeon SCGCAAA259B11
candidate divison MSBL1 archaeon SCGCAAA259D14
candidate divison MSBL1 archaeon SCGCAAA259E17
candidate divison MSBL1 archaeon SCGCAAA259E19
candidate divison MSBL1 archaeon SCGCAAA259E22
candidate divison MSBL1 archaeon SCGCAAA259I09
candidate divison MSBL1 archaeon SCGCAAA259I14
candidate divison MSBL1 archaeon SCGCAAA259J03
candidate divison MSBL1 archaeon SCGCAAA259M10
candidate divison MSBL1 archaeon SCGCAAA259O05
candidate divison MSBL1 archaeon SCGCAAA261F17
candidate divison MSBL1 archaeon SCGCAAA382A13
candidate divison MSBL1 archaeon SCGCAAA382A20
candidate divison MSBL1 archaeon SCGCAAA382F02
candidate divison MSBL1 archaeon SCGCAAA833F18
Candidatus Aenigmarchaeota archaeon CG1_02_38_14
Candidatus Aenigmarchaeota archaeon JGI 0000106F11
Candidatus Bathyarchaeota archaeon B261
Candidatus Bathyarchaeota archaeon TCS64
Candidatus Haloredivivus sp. G17
Candidatus Heimdallarchaeota archaeon LC_3
Candidatus Korarchaeum cryptofilum OPF8
Candidatus Lokiarchaeota archaeon CR_4
Candidatus Micrarchaeum acidiphilum ARMAN2
Candidatus Nanopusillus acidilobi
Candidatus Nanosalina sp. J07AB43
Candidatus Nanosalinarum sp. J07AB56
Candidatus Thorarchaeota archaeon SMTZ145
DHVEG6 Guaymas35
Euryarchaeota archaeon SCGC AAA286E23
Ferroglobus placidus DSM 10642
Geoglobus acetivorans
Geoglobus ahangari
Hadesarchaea archaeon DG33
Hadesarchaea archaeon DG331
Hadesarchaea archaeon YNP_45
Hadesarchaea archaeon YNP_N21
Halococcus sediminicola
Lokiarchaeum sp. GC14_75
Methanobacteriaceae archaeon 41_258
Methanobacterium arcticum
Methanobacterium formicicum
Methanobacterium lacus
Methanobacterium paludis
Methanobacterium sp. 42_16
Methanobacterium sp. Maddingley MBC34
Methanobacterium sp. MB1
Methanobacterium sp. SMA27
Methanobacterium veterum
Methanobrevibacter arboriphilus ANOR1
Methanobrevibacter boviskoreani JH1
Methanobrevibacter millerae
Methanobrevibacter olleyae
Methanobrevibacter oralis JMR01
Methanobrevibacter ruminantium M1
Methanobrevibacter smithii ATCC 35061
Methanobrevibacter smithii CAG186
Methanobrevibacter sp. AbM4
Methanobrevibacter wolinii SH
Methanocaldococcus bathoardescens
Methanocaldococcus fervens AG86
Methanocaldococcus jannaschii DSM 2661
Methanocaldococcus sp. FS40622
Methanocaldococcus villosus KIN24T80
Methanocaldococcus vulcanius M7
Methanocella arvoryzae MRE50
Methanocella conradii HZ254
Methanocella paludicola SANAE
Methanococcus aeolicus Nankai3
Methanococcus maripaludis C7
Methanococcus vannielii SB
Methanococcus voltae A3
Methanoculleus bourgensis MS2
Methanoculleus chikugoensis JCM 10825
Methanoculleus marisnigri JR1
Methanoculleus sediminis
Methanoculleus sp. DTU007
Methanoculleus sp. MH98A
Methanoflorens stordalenmirensis
Methanofollis liminatans DSM 4140
Methanolinea sp. SDB
Methanolinea tarda NOBI1
Methanomicrobium mobile BP
Methanoregula formicica SMSP
Methanosphaera stadtmanae DSM 3091
Methanospirillum hungatei JF1
Methanothermobacter marburgensis str. Marburg
Methanothermobacter sp. CaT2
Methanothermobacter thermautotrophicus str. Delta H
Methanothermococcus okinawensis IH1
Methanothermococcus thermolithotrophicus DSM 2095
Methanothermus fervidus DSM 2088
Methanotorris formicicus McS70
Methanotorris igneus Kol 5
Nanohaloarchaea archaeon AB578D14
Nanohaloarchaea archaeon B1Br10_U2g1
Nanohaloarchaea archaeon B1Br10_U2g19
Nanohaloarchaea archaeon B1Br10_U2g21
Nanohaloarchaea archaeon B1Br10_U2g29
Nanohaloarchaea archaeon PLBr10_U2g16
Nanohaloarchaea archaeon PLBr10_U2g19
Nanohaloarchaea archaeon PLBr10_U2g27
Nitrosopumilus sp. AR
Palaeococcus ferrophilus DSM 13482
Palaeococcus pacificus DY20341
Pyrococcus abyssi GE5
Pyrococcus furiosus DSM 3638
Pyrococcus horikoshii OT3
Pyrococcus sp. NA2
Pyrococcus sp. ST04
Thermococcus barophilus MP
Thermococcus celer JCM 8558
Thermococcus celericrescens
Thermococcus cleftensis
Thermococcus eurythermalis
Thermococcus gammatolerans EJ3
Thermococcus kodakarensis KOD1
Thermococcus litoralis DSM 5473
Thermococcus nautili
Thermococcus onnurineus NA1
Thermococcus paralvinellae
Thermococcus sibiricus MM 739
Thermococcus sp. 2319x1
Thermococcus sp. 40_45
Thermococcus sp. 4557
Thermococcus sp. AM4
Thermococcus sp. EP1
Thermococcus sp. JCM 11816
Thermococcus sp. PK
Thermococcus zilligii AN1
uncultured marine crenarchaeote 'Gulf of Maine'
10
16
19
20
49
50
52
53
54
59
Residue position (relative to HMfB sequence)
Number of histones with subtitution at each position
0
1
2
3
4
5
6

Figure S9. Phyletic distribution of excess amino acid diversity at selected structurally sensitive sites in archaea with more than one histone. Substitutions are defined as amino acids which differ from the most commonly found amino acid at that position. Species are grouped into higher order classification.

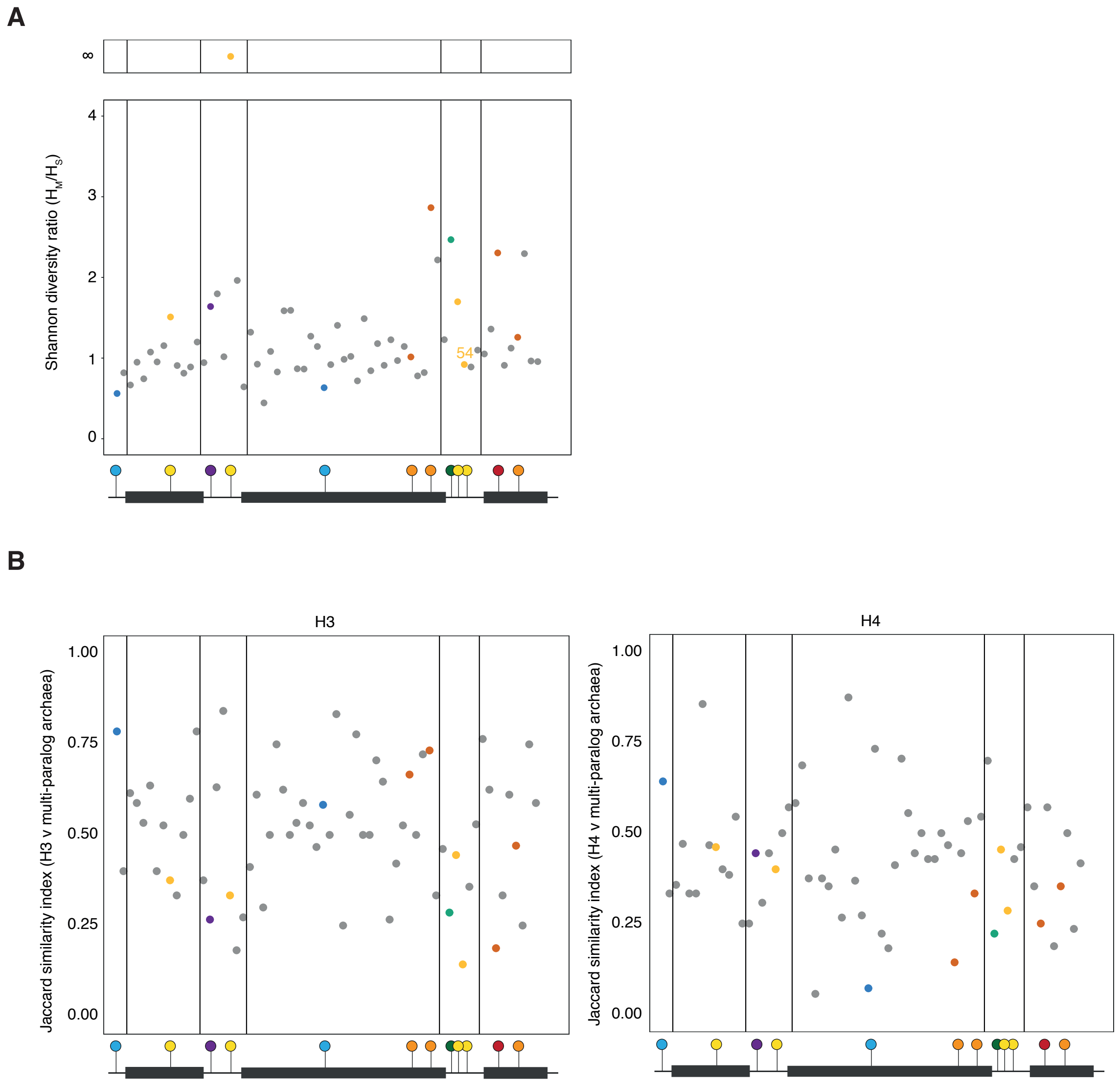

Figure S10. (A) Shannon diversity ratio (H_M/H_S) across the histone fold excluding Asgard archaea. Note the precipitous drop in the Shannon ratio at residue 54 compared to Figure 7. (B) Jaccard similarity at each position in the core histone fold domain comparing archaea with more than one histone gene to H3 (left) and H4 (right).

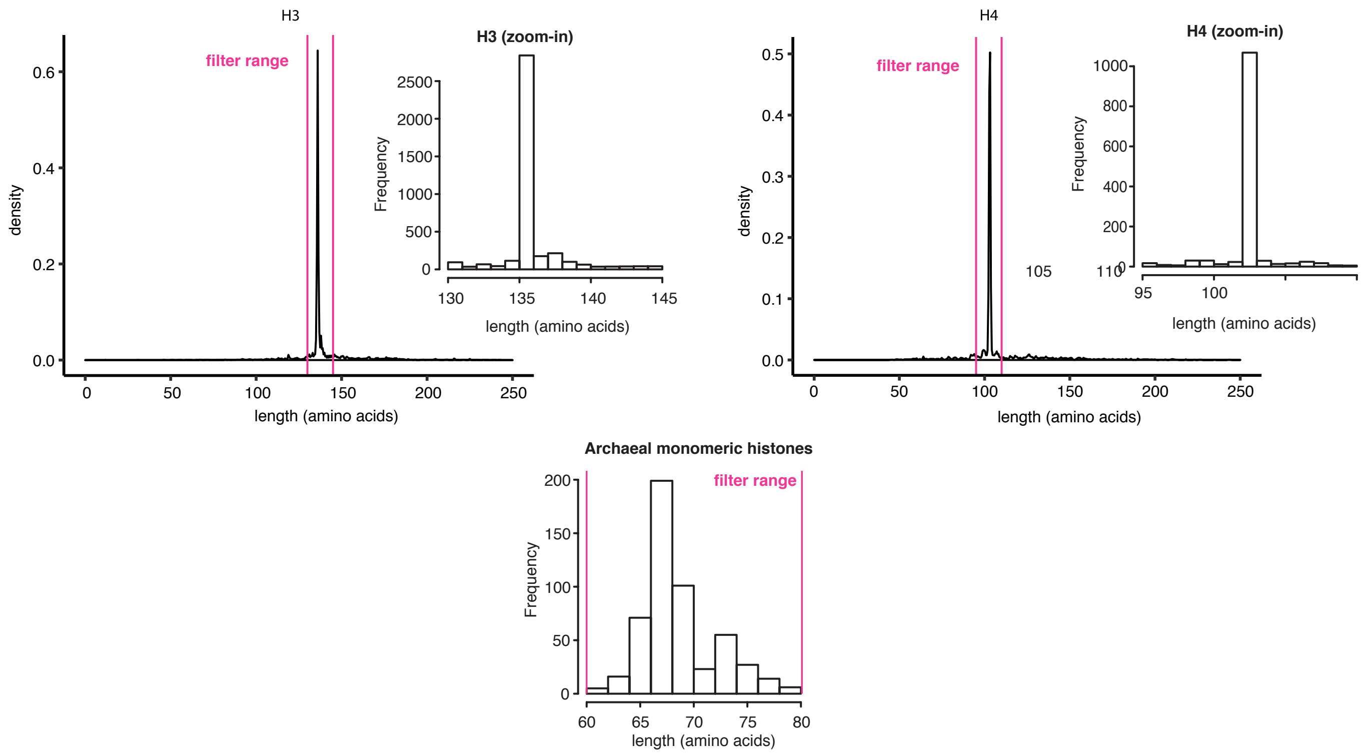

Figure S11. Length distribution of putative H3 and H4 proteins prior to filtering. Size thresholds applied are highlighted in pink. The distribution of histone length in archaea after filtering is given below.

Table S1. Genomes with candidate capstones

Species	Amino acid property	Amino acid at position 49	Protein ID(s)
archaeon GW2011-AR5	hydrophobic	F	KHO48101.1
candidate division MSBL1 archaeon SCGC-AAA259D14	negative	E	KXA88638.1
candidate division MSBL1 archaeon SCGC-AAA259E17	hydrophobic	A	KXA92862.1
candidate division MSBL1 archaeon SCGC-AAA259E22	negative	E	KXA93729.1
candidate division MSBL1 archaeon SCGC-AAA259O05	hydrophobic	A	KXA99006.1
candidate division MSBL1 archaeon SCGC-AAA261G05	hydrophobic	A	KXB04110.1
candidate division MSBL1 archaeon SCGC-AAA382F02	hydrophobic	A	KXB06501.1
candidate division MSBL1 archaeon SCGC-AAA833F18	hydrophobic	A	KXB09675.1
candidate division MSBL1 archaeon SCGC-AAA833K04	hydrophobic	A	KXB09405.1
Candidatus Caldiarchaeum subterraneum	hydrophobic	Y	BAJ51262.1
Candidatus Haloredivivus sp. G17	hydrophobic	F	EHK00678.1
Candidatus Heimdallarchaeota archaeon LC_3	hydrophobic	M	OLS18443.1
Candidatus Nanosalina sp. J07AB43	negative	D	EGQ43804.1
Candidatus Nanosalinarum sp. J07AB56	negative	D	EGQ40276.1
DHVEG-6 Guayamas35	hydrophobic	Y	D1191683417049<br>D11977717971
Hadesarchaea archaeon DG-33	hydrophobic	I	KUO39119.1
Hadesarchaea archaeon DG-33-1	hydrophobic	I	KUO41575.1
Hadesarchaea archaeon YNP_N21	hydrophobic	A	KUO43356.1
Methanobrevibacter boviskoreani JH1	hydrophobic	I	WP_040681874.1
Methanobrevibacter millerae	hydrophobic	L	ALT67815.1
Methanobrevibacter olleyae	hydrophobic	I<br>L	AMK15744.1<br>AMK16160.1
Methanobrevibacter oralis JMR01	hydrophobic	I	WP_042694673.1
Methanobrevibacter ruminantium M1	hydrophobic	I<br>M	ADC47581.1<br>ADC45863.1
Methanobrevibacter smithii ATCC 35061	hydrophobic	I	ABQ87465.1
Methanobrevibacter sp. AbM4	hydrophobic	I	AGN16739.1
Methanobrevibacter wolinii SH	hydrophobic	L	WP_042707783.1
Methanocaldococcus villosus KIN24-T80	negative	E	WP_004593351.1

Methanosphaera stadtmanae DSM 3091	negative	E	ABC56784.1
miscellaneous Crenarchaeota group-15 archaeon DG-45	negative	E	KON30096.1
Nanohaloarchaea archaeon B1-Br10_U2g1	negative	D	N4531936719564
Nanohaloarchaea archaeon B1-Br10_U2g21	negative<br>hydrophobic	E<br>Y	N45586728869<br>N4559769497894
Nanohaloarchaea archaeon B1-Br10_U2g29	negative<br>hydrophobic	E<br>Y	N4561231812515<br>N4562282223022
Nanohaloarchaea archaeon PL-Br10_U2g16	negative	E	N6651626116458
Nanohaloarchaea archaeon PL-Br10_U2g19	negative	D<br>E	N6641176711964<br>N66436133849
Nanohaloarchaea archaeon PL-Br10_U2g27	negative<br>hydrophobic	E<br>Y	N6661406114258<br>N66662046404

Table S2. HMfB single amino acid mutants characterised in gel shift assays in (2)

Mutation	ΔΔG DNA binding (kcal/mol)	ΔΔG stability of the protein (kcal/mol)	Gel shift result from Soares *et al.* (2000)
M1G	-0.229	2.373	Same gel shift as WT
E2D	-1.5081	0.725	Decreased gel shift
E2K	-1.8864	-17.952	Same gel shift
L3C	-0.866	12.1636	Did not accumulate in E. coli
L3I	-0.4955	0.234	Decreased gel shift
P4C	-0.7506	15.609	Same gel shift
P4S	3.5532	14.2371	Same gel shift
I5V	-1.0162	3.758	Same gel shift
A6P	30.558863	-3.104	Did not accumulate in E. coli
P7A	1.4649	13.8786	Same gel shift
I8V	0.0469	4.354	Same gel shift
G9D	0.9748	0.818	Did not accumulate in E. coli
R10S	1.41	0.315	Did not accumulate in E. coli
R10K	1.8425	-6.518	Decreased gel shift
R10G	1.4124	0.416	Did not accumulate in E. coli
I11L	-0.7244	-4.562	Same gel shift
K13E	-0.2283	-4.133	Did not accumulate in E. coli
K13Q	-0.4862	-1.443	Did not accumulate in E. coli
K13T	-0.9661	4.569	Did not accumulate in E. coli
K13R	-2.0568	-1.117	Same gel shift
D14E	-0.3723	-5.831	Did not accumulate in E. coli
D14N	-0.493	-9.57	Same gel shift
D14K	-1.24	-16.484	Decreased gel shift
D14H	-3.0588	-14.311	Did not accumulate in E. coli
A15S	0	7.3696	Same gel shift
A15G	0	7.3371	Did not accumulate in E. coli
E18D	1.2779	0.034	Same gel shift
E18P	0.4728	-5.265	Same gel shift
E18K	-1.4242	3.089	Did not accumulate in E. coli
R19I	4.3091	-3.551	Did not accumulate in E. coli
R19Q	5.2768	-2.048	Did not accumulate in E. coli
R19S	9.9084	1.502	Did not accumulate in E. coli
R19G	10.5705	5.8858	Decreased gel shift
R19K	5.6178	-1.358	Decreased gel shift
V20C	-0.4124	9.6386	Did not accumulate in E. coli
V20I	0.3076	-6.426	Same gel shift
V20D	-1.2659	19.0681	Did not accumulate in E. coli
S21A	1.9393	1.831	Decreased gel shift
S21C	-1.3203	7.5993	Same gel shift
S21T	1.5381	-1.531	Decreased gel shift
R25K	5.5543	-6.126	Same gel shift
I26E	0.3859	5.25	Decreased gel shift
T27A	-0.0002	3.174	Same gel shift
T27C	0	6.5756	Same gel shift
I31V	0.0005	6.4488	Same gel shift
I31C	-0.0019	15.9234	Same gel shift
L32C	0	19.2103	Did not accumulate in E. coli
M35C	0	10.6139	Same gel shift
R37K	-0.1871	-8.6	Same gel shift
R37L	0.024	-4.509	Decreased gel shift
R37Q	-0.014	2.771	Decreased gel shift
D38E	0	-3.054	Same gel shift
I39V	0	1.634	Same gel shift
I39C	0	11.2149	Same gel shift
A43I	0.0061	32.1119	Did not accumulate in E. coli
S41C	0.012	0.715	Same gel shift
I44V	0	3.902	Same gel shift
L46I	0.0158	6.3608	Decreased gel shift
L46V	0.0079	10.9782	Decreased gel shift
L46F	-0.0037	4.347	Same gel shift
L46S	0.0021	20.4698	Did not accumulate in E. coli
R48K	0.3128	-0.278	Same gel shift
H49A	0.1917	1.99	Decreased gel shift
H49D	0.5219	11.2511	Same gel shift
G51K	-0.9888	8.6454	Did not accumulate in E. coli
G51A	-0.677	11.4035	Did not accumulate in E. coli
R52K	-0.252	1.341	Decreased gel shift
R52A	1.4069	5.407	Did not accumulate in E. coli
R52H	4.0269	5.6672	Did not accumulate in E. coli
R52Q	-0.5464	3.51	Did not accumulate in E. coli
K53R	1.6919	-1.096	Decreased gel shift
K53E	3.2454	1.266	Did not accumulate in E. coli
K53T	2.1058	2.286	Did not accumulate in E. coli
T54A	3.5459	2.269	Did not accumulate in E. coli
T54C	2.7237	1.158	Did not accumulate in E. coli
T54K	4.9682	1.889	Did not accumulate in E. coli
T54R	7.0644	4.545	Did not accumulate in E. coli
T54S	2.2691	4.55	Did not accumulate in E. coli
T54V	-1.0832	-2.153	Did not accumulate in E. coli
T54Y	19.2299	19.3918	Did not accumulate in E. coli
I55V	0.662	4.123	Same gel shift
I55L	0.2032	-0.262	Decreased gel shift
I55T	0.6911	17.92	Decreased gel shift
I55M	-0.2553	4.661	Decreased gel shift
I55C	0.285	16.4504	Did not accumulate in E. coli
K56R	-1.1339	0.905	Same gel shift
K56T	-1.529	2.93	Did not accumulate in E. coli
K56E	2.0138	-1.73	Did not accumulate in E. coli
K56I	-0.6459	4.121	Did not accumulate in E. coli
K56Q	-1.3688	0.836	Did not accumulate in E. coli
E58S	-1.7313	5.415	Did not accumulate in E. coli
D59A	1.3352	9.3873	Did not accumulate in E. coli
D59E	-1.5257	5.7071	Did not accumulate in E. coli
D59N	-0.1062	13.877	Did not accumulate in E. coli
I60V	0	4.055	Same gel shift
E61A	0.006	1.404	Same gel shift
E61V	-0.0047	3.984	Same gel shift
E61Q	0.0064	-1.091	Same gel shift
E61K	0.0129	-2.887	Decreased gel shift
E61R	-0.0027	-2.443	Decreased gel shift
L62I	0.0099	5.129	Decreased gel shift
L62V	0.007	7.5351	Decreased gel shift
L62Y	-0.096	3.525	Same gel shift
L62M	0.0173	-2.076	Did not accumulate in E. coli
V64R	-0.0057	4.251	Decreased gel shift
R65K	-0.0062	2.925	Same gel shift
R66M	0.0066	-1.184	Same gel shift

Table S3. HMfB and *S. cerevisiae* H3/H4 sequences and residue numbering for each position in the alignment used for diversity analysis

M. fervidus HMfB residue number	*M. fervidus* HMfB residue	*S. cerevisiae* H3 residue number (P61830)	*S. cerevisiae* H3 residue	*S. cerevisiae* H4 residue number (P02309)	*S. cerevisiae* H4 residue
2	E	60	E	29	G
3	L	63	I	30	I
4	P	64	R	31	T
5	I	65	K	32	K
6	A	66	L	33	P
7	P	67	P	34	A
8	I	68	F	35	I
9	G	69	Q	36	R
10	R	70	R	37	R
11	I	71	L	38	L
12	I	72	V	39	A
13	K	73	R	40	R
14	D	74	E	41	R
15	A	75	I	42	G
16	G	76	A	43	G
17	A	77	Q	44	V
18	E	78	D	45	K
19	R	84	R	46	R
20	V	85	F	47	I
21	S	86	Q	48	S
22	D	87	S	49	G
23	D	88	S	50	L
24	A	89	A	51	I
25	R	90	I	52	Y
26	I	91	G	53	E
27	T	92	A	54	E
28	L	93	L	55	V
29	A	94	Q	56	R
30	K	95	E	57	A
31	I	96	S	58	V
32	L	97	V	59	L
33	E	98	E	60	K
34	E	99	A	61	S
35	M	100	Y	62	F
36	G	101	L	63	L
37	R	102	V	64	E
38	D	103	S	65	S
39	I	104	L	66	V
40	A	105	F	67	I
41	S	106	E	68	R
42	E	107	D	69	D
43	A	108	T	70	S
44	I	109	N	71	V
45	K	110	L	72	T
46	L	111	A	73	Y
47	A	112	A	74	T
48	R	113	I	75	E
49	H	114	H	76	H
50	A	115	A	77	A
51	G	116	K	78	K
52	R	117	R	79	R
53	K	118	V	80	K
54	T	119	T	81	T
55	I	120	I	82	V
56	K	121	Q	83	T
57	A	122	K	84	S
58	E	123	K	85	L
59	D	124	D	86	D
60	I	125	I	87	V
61	E	126	K	88	V
62	L	127	L	89	Y
63	A	128	A	90	A
64	V	129	R	91	L
65	R	130	R	92	K

Table S4: Primers used for qRT-PCR

Target	Primer sequence forward (5’-3’)	Primer sequence revers (5’-3’)
Msp_0122	CAATTGCTCCTGTAGGCAGA	TACTGCATCTTCTGCGATGG
Msp_0168	TGCTGGAGCAGACAGAATCA	TTTTCTTCCTGCATGTTTTGC
Msp_0383	AACGGTGGTGCAGAAAGAGT	TTTCTTCCATTTTCTTCAGCATT
Msp_0518	GGAGCAGCACGTGTAAGTGA	CTGCGTGTTTTGCAAGAAGT
Msp_0614	GGTGCACCTAGAGCAACAAA	TCAACATCAGATGCTTTCACTG
Msp_0769	AAAAGCAACAATGGGTCGTT	AAAGGCACTGCATCTCTTCC
Msp_0924.5	TGACAGAAATACCAAAGCACCT	TGCGGATGCTAATTTGTCAG
Msp_16S	AGGAGCGACAGCAGAATGAT	CAGGACGCTTCACAGTACGA
Msp_rpoB	TGCTTGGTATTTGTGCTGGA	TCCAAGAGCCTGTTTTGTCA

References

1. Borrel G, Brugere J-F, Gribaldo S, Schmitz RA, Moissl-Eichinger C (2020) The host-associated archaeome. Nat Rev Microbiol 18(11):622-636.
2. Soares DJ, Sandman K, Reeve JN (2000) Mutational analysis of archaeal histone-DNA interactions. Journal of Molecular Biology 297(1):39–47.
